# Evolution, diversity, and disparity of the tiger shark lineage *Galeocerdo* in deep time

**DOI:** 10.1017/pab.2021.6

**Published:** 2021-03-24

**Authors:** Julia Türtscher, Faviel A. López-Romero, Patrick L. Jambura, René Kindlimann, David J. Ward, Jürgen Kriwet

**Affiliations:** Department of Palaeontology, University of Vienna, Althanstraβe 14, 1090 Vienna, Austria; Department of Palaeontology, University of Vienna, Althanstraβe 14, 1090 Vienna, Austria; Department of Palaeontology, University of Vienna, Althanstraβe 14, 1090 Vienna, Austria; Haimuseum und Sammlung R. Kindlimann, Aathal-Seegräben, Switzerland; Department of Earth Sciences, Natural History Museum, Cromwell Road, London SW7 5BD, U.K.; Department of Palaeontology, University of Vienna, Althanstraβe 14, 1090 Vienna, Austria

## Abstract

Sharks have a long and rich fossil record that consists predominantly of isolated teeth due to the poorly mineralized cartilaginous skeleton. Tiger sharks (*Galeocerdo*), which represent apex predators in modern oceans, have a known fossil record extending back into the early Eocene (ca. 56 Ma) and comprise 22 recognized extinct and one extant species to date. However, many of the fossil species remain dubious, resulting in a still unresolved evolutionary history of the tiger shark genus. Here, we present a revision of the fossil record of *Galeocerdo* by examining the morphological diversity and disparity of teeth in deep time. We use landmark-based geometric morphometrics to quantify tooth shapes and qualitative morphological characters for species discrimination. Employing this combined approach on fossil and extant tiger shark teeth, our results only support six species to represent valid taxa. Furthermore, the disparity analysis revealed that diversity and disparity are not implicitly correlated and that *Galeocerdo* retained a relatively high dental disparity since the Miocene despite its decrease from four to one species. With this study, we demonstrate that the combined approach of quantitative geometric morphometric techniques and qualitative morphological comparisons on isolated shark teeth provides a useful tool to distinguish between species with highly similar tooth morphologies.

## Introduction

Sharks, rays, and skates belong to the cartilaginous fish (Chondrichthyes), forming a monophyletic group, the Neoselachii sensu Compagno (1977) or Elasmobranchii sensu Maisey (2012). So far, more than 500 species of sharks have been described, which amounts to more than 40% of all known extant chondrichthyans (Ebert et al. 2013; Weigmann 2016). Although they are low in number compared with bony fishes, sharks represent a diverse group, occupying many different ecological niches and trophic levels—from zooplanktivorous species at low trophic levels (e.g., whale shark *Rhincodon typus*), to secondary consumers (e.g., nurse shark *Ginglymostoma cirratum*), and third-order consumers (e.g., white shark *Carcharodon carcharias* and tiger shark *Galeocerdo cuvier*), which is similar to marine mammals (Cortès 1999). They occur in both marine and freshwater environments, ranging from the surface to more than 4000 m depth and are distributed worldwide, from arctic waters (down to 0.6°C water temperature) to tropical waters (Ebert et al. 2013). Therefore, this group, which survived several mass extinction events since its first appearance in the fossil record, can be considered very successful despite the rather small taxonomic diversity.

Despite their obvious evolutionary and ecological success, an increasing number of shark species are threatened with extinction as a result of overfishing, habitat degradation, and climate change. Currently we are facing an anthropogenically driven mass extinction event, very similar to past mass extinctions, with global extinction rates being elevated up to a thousand times higher than former extinction events as shown by the fossil record (Pimm et al. 1995; Ceballos et al. 2017). Compared with most other vertebrates, the extinction risk for chondrichthyans is substantially higher, large-bodied and shallow-water species are especially at greatest risk, and overall, only one-third of all chondrichthyan species can be regarded as safe (Dulvy et al. 2014; Stein et al. 2018).

While several iconic sharks such as the white shark attracted a large proportion of scientific interest in recent years, the tiger shark and its evolutionary history remained largely ignored. Due to their tremendous size of up to 5.5m (Holmes et al. 2012), their broad and heavily mineralized jaws (Moss 1972), and their specialized teeth, tiger sharks are capable of preying on a wide variety of different food items, including sea turtles, birds, marine mammals, and even garbage (Gudger 1949; Compagno 1984; Randall 1992; Kent 2018).

**Figure 1:**
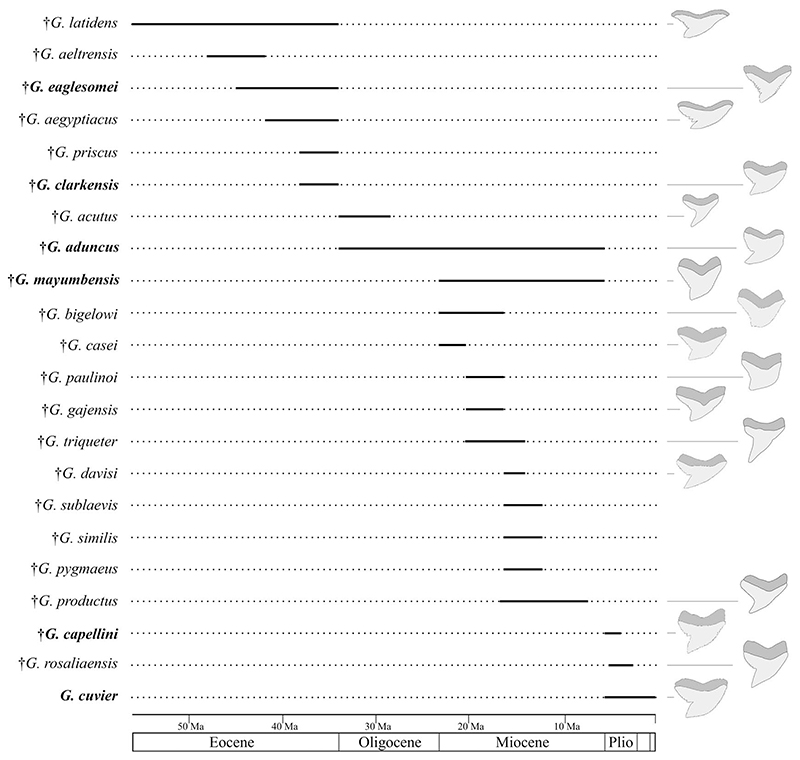
First and last occurrence of nominal species of the genus *Galeocerdo* (see Supplementary Material). †*Galeocerdo subcrenatus* is not listed, as no formation is indicated in the original description (see Emmons 1858). The species considered valid in this study are written in bold.

A well-founded knowledge of the fossil record of *Galeocerdo* and its nominal species is important for diversity and disparity analyses through geologic time to better understand how vulnerable the modern tiger shark is to current threats. It is, however, represented entirely by isolated teeth, because the skeleton consists predominantly of cartilage, which fossilizes only under very specific conditions, rendering the identification of fossil taxa difficult. The oldest occurrences of tiger sharks were reported from the Ypresian, early Eocene (ca. 56–47.8 Ma; Cappetta 1981, 1987). In the last 200 years, more than 60 fossil *Galeocerdo* species were described based on isolated teeth and even scales, many of which meanwhile were reassigned to other genera and species. This resulted in 23 valid species, some of which are considered doubtful, depending on authors (see Supplementary Material). Based on these records, an increase in diversity from the Paleogene (Eocene–Oligocene with 8 species) to the Neogene–Quaternary (Miocene–Holocene with 15 species) seemingly occurred, before all but 1 species went extinct in the late Miocene to Pliocene (Fig. 1).

The high morphological similarity of extinct tiger shark teeth led to a poorly understood evolutionary history of this unique genus, which is of great ecological importance. The goal of the present study is to revise the fossil record of the extant *G*. *cuvier* and the extinct members of the tiger shark lineage by applying geometric morphometric techniques. Furthermore, teeth of *Galeocerdo* are compared with genera exhibiting superficially similar-looking teeth, that is, *Hemipristis* and †*Physogaleus*, to validate the applicability of this method to separate similar-appearing teeth of different genera.

Significant morphological inter/intraspecific variations and similarities between teeth of extinct and extant tiger shark species were examined and were used in combination with qualitative (morphological) characters to evaluate and establish the validity of extinct tiger shark species and to revise the fossil record of the extant species. Additionally, short morphological descriptions of the 23 previously recognized extinct and extant tiger shark species are provided (see Supplementary Material).

## Material and Methods

### Material

Isolated teeth of extinct and extant shark species as well as dried jaws of the extant tiger shark *Galeocerdo cuvier* were used in this study. A dagger preceding the name identifies extinct species in the text as well as in all tables and figures.

The sample consists in total of 569 shark teeth, photographed in labial view. Eighteen published illustrations of tiger shark teeth were used if insufficient or no other material of the corresponding species was available. The majority of the teeth (*n* = 450) belongs to tiger sharks and is represented by the 16 nominal species: †*G*. *acutus*, †*G*. *aduncus*, †*G*. *aegyptiacus*, †*G*. *bigelowi*, †*G*. *capellini*, †*G*. *casei*, †*G*. *clarkensis*, *G*. *cuvier*, †*G*. *davisi*, †*G*. *eaglesomei*, †*G*. *gajensis*, †*G*. *latidens*, †*G*. *mayumbensis*, †*G*. *paulinoi*, †*G*. *rosaliaensis*, and †*G*. *triqueter*. However, teeth of morphologically similar species of *Hemipristis* (†*H*. *curvatus* and †*H*. *serra*) and †*Physogaleus* (†*P*. *alabamensis* and †*P*. *contortus*) were also included. Five species were represented with their holotype (†*G*. *casei*, †*G*. *davisi*, †*G*. *gajensis*, †*G*. *paulinoi*, †*P*. *alabamensis*) and three with the whole type series (†*G*. *clarkensis*, †*G*. *eaglesomei*, †*G*. *rosaliaensis*). Detailed information about the material (e.g., provenance, geologic age) is depicted in Supplementary Table 1.

### Geometric Morphometrics

**Figure 2:**
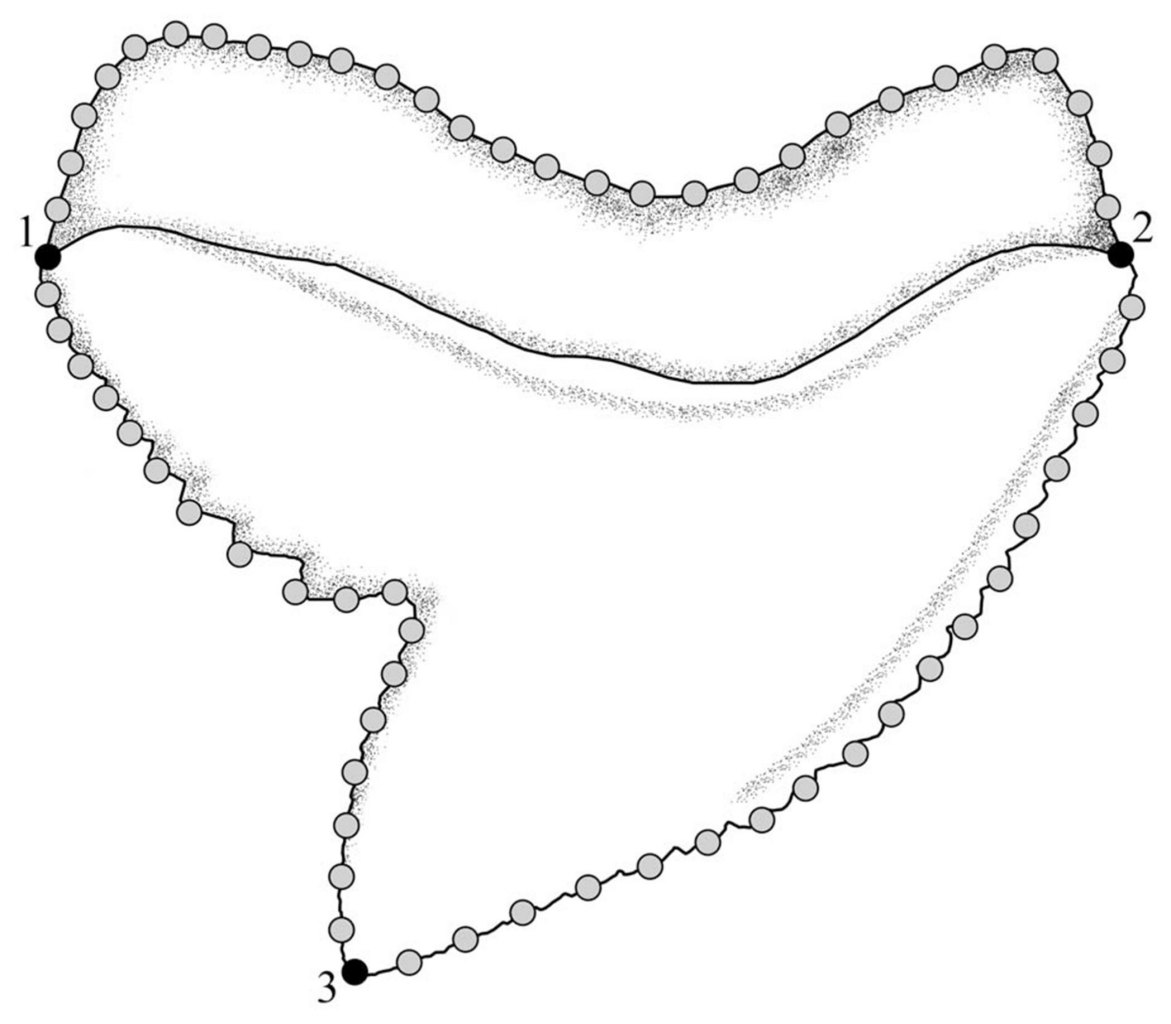
Location of the landmarks and semilandmarks for the geometric morphometric analyses. The landmarks are located on the (1) base of distal cutting edge, (2) base of mesial cutting edge, and (3) tip of cusp. Twenty-eight semilandmarks are located along the outline of the root between the base of the distal and mesial cutting edge, 18 are located between the base of the mesial cutting edge, and the tip of the cusp and 18 semilandmarks are situated between the tip of the cusp and the base of the distal cutting edge.

The tooth shape of fossil and extant *Galeocerdo* species as well as *Hemipristis* and †*Physogaleus* was studied with 2D landmark-based geometric morphometrics. Three homologous landmarks were digitized using the software tpsDIG2 (v. 2.31; Rohlf 2017). Additionally, 64 semilandmarks were digitized between the homologous landmarks to capture the overall tooth shape (Fig. 2).

To minimize the variance caused by size, orientation, location, and rotation, a generalized Procrustes analysis (GPA) was performed on the landmark coordinates. The sliding semilandmarks were allowed to slide to minimize the bending energy (Gunz and Mitteroecker 2013). The aligned coordinates were then subjected to a principal component analysis (PCA) to assess shape variation of teeth. Tooth shape differences between genera and within *Galeocerdo* species were estimated with a permutational analysis of variance (ANOVA), followed by pairwise comparisons between the groups, with the functions *procD.lm* and *pairwise* considering the distances between means in the R packages geomorph (v. 3.1; Adams et al. 2016) and RRPP (Collyer and Adams 2018).

### Disparity through Time

To evaluate how the dental morphological disparity through geologic time changed among *Galeocerdo* species, we assigned taxa to the time bins Eocene, Oligocene, Miocene, Pliocene, and Holocene. We used the Procrustes variance (Zelditch et al. 2012), applied the function *morphol*.*disparity* in the R package geomorph (v. 3.1; Adams et al. 2016), and performed post hoc pairwise comparisons considering the variance between the epochs to estimate differences between them.

### Qualitative Reevaluation of Morphological Characters

Although geometric morphometrics is a useful method to quantify shape and morphology, some qualitative characters are not taken into account with this technique. In shark teeth, such features include the presence of serrations on the cutting edges as well as their quality (e.g., coarse, compound, minor), characters that are often essential for identifications on the species level. To enable a best possible determination of the teeth included in the present study, all species were examined and described, incorporating diagnostic characters from first descriptions and additional literature (see Supplementary Material). The tooth terminology used broadly follows Cappetta (2012).

## Results

### PCA on the Whole Sample

**Figure 3:**
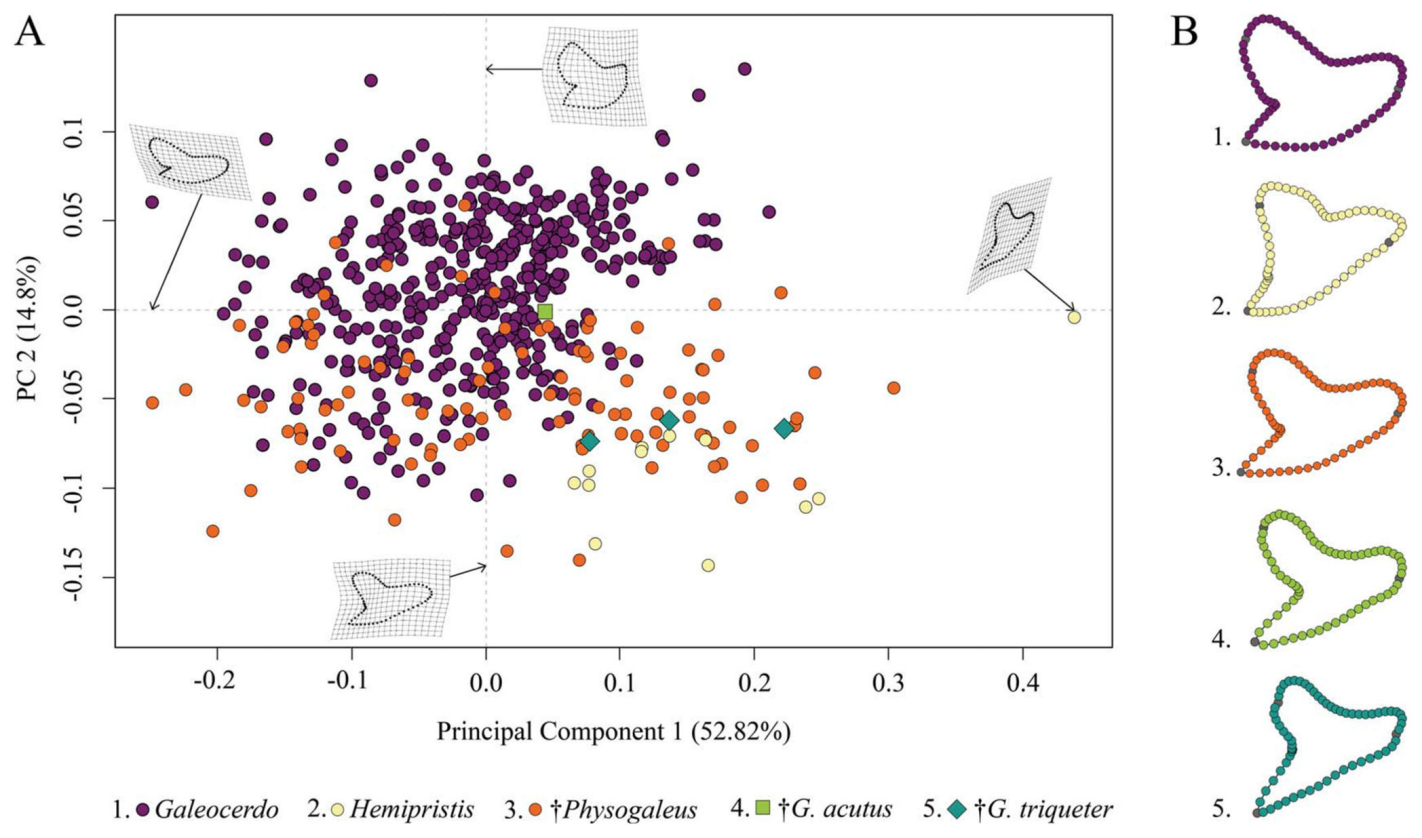
Morphospace of all examined shark teeth, divided into the three genera *Galeocerdo*, *Hemipristis*, and †*Physogaleus*, with the two species †*G*. *acutus* and †*G*. *triqueter* highlighted. A, Scatter plot of the first two principal component (PC) axes. B, Mean tooth shapes of all examined groups.

This analysis contained a total of 569 specimens, representing the three genera *Galeocerdo*, *Hemipristis*, and †*Physogaleus* (see Supplementary Table 1) and resulted in 134 PC axes with the first four explaining 83.9% of the total morphological variation. All remaining axes each account for less than 5% of the variance. PC 1 (52.82%) describes a morphological shift from broad and labiolingually compressed teeth with a flattened root and a strongly distally inclined cusp on the negative side to high teeth having a V-shaped root and nearly vertical distal and mesial cutting edges and lacking a distinct distal notch on the positive side. Positive scores of PC 2 (14.8%) are related to very broad and bulky teeth with an asymmetrical root, a strongly curved mesial cutting edge, a deep distal notch, and a convex distal heel. Negative scores indicate slenderer teeth with a symmetrical root, a less-curved mesial cutting edge, a more obtuse-angled notch, and a straight distal heel (Fig. 3).

The morphospace occupied by *Hemipristis* is completely separated from *Galeocerdo*, whereas the morphospace of †*Physogaleus* overlaps with both *Galeocerdo* and *Hemipristis*.

Two doubtful species, the Oligocene †*G*. *acutus* and the Miocene †*G*. *triqueter* (represented by one and three teeth, respectively), were included to determine their genus affiliations. According to our results, †*G*. *acutus* plots within the overlapping area of †*Physogaleus* and *Galeocerdo*, while †*G*. *triqueter* is located exclusively within the morphospace of †*Physogaleus* (Fig. 3). Morphological similarities of both species with †*Physogaleus contortus* include slender and smooth/minor serrated tooth crowns and bulky roots, supporting their assignment to the genus †*Physogaleus* (Fig. 3B, Supplementary Material).

The implemented permutational ANOVA (PERMANOVA) reveals significant differences in shape among *Galeocerdo*, *Hemipristis*, and †*Physogaleus* (Table 1). A pairwise comparison further corroborates the separation of all three genera (Table 2).

### Tiger Shark Species Shape Variation

This analysis contained a total of 450 *Galeocerdo* specimens, assigned to the nominal species †*G*. *aduncus*, †*G*. *bigelowi*, †*G*. *capellini*, †*G*. *casei*, †*G*. *clarkensis*, *G*. *cuvier*, †*G*. *davisi*, †*G*. *eaglesomei*, †*G*. *gajensis*, †*G*. *latidens*, †*G*. *mayumbensis*, †*G*. *paulinoi* and †*G*. *rosaliaensis* (see Supplementary Table 1).

**Figure 4:**
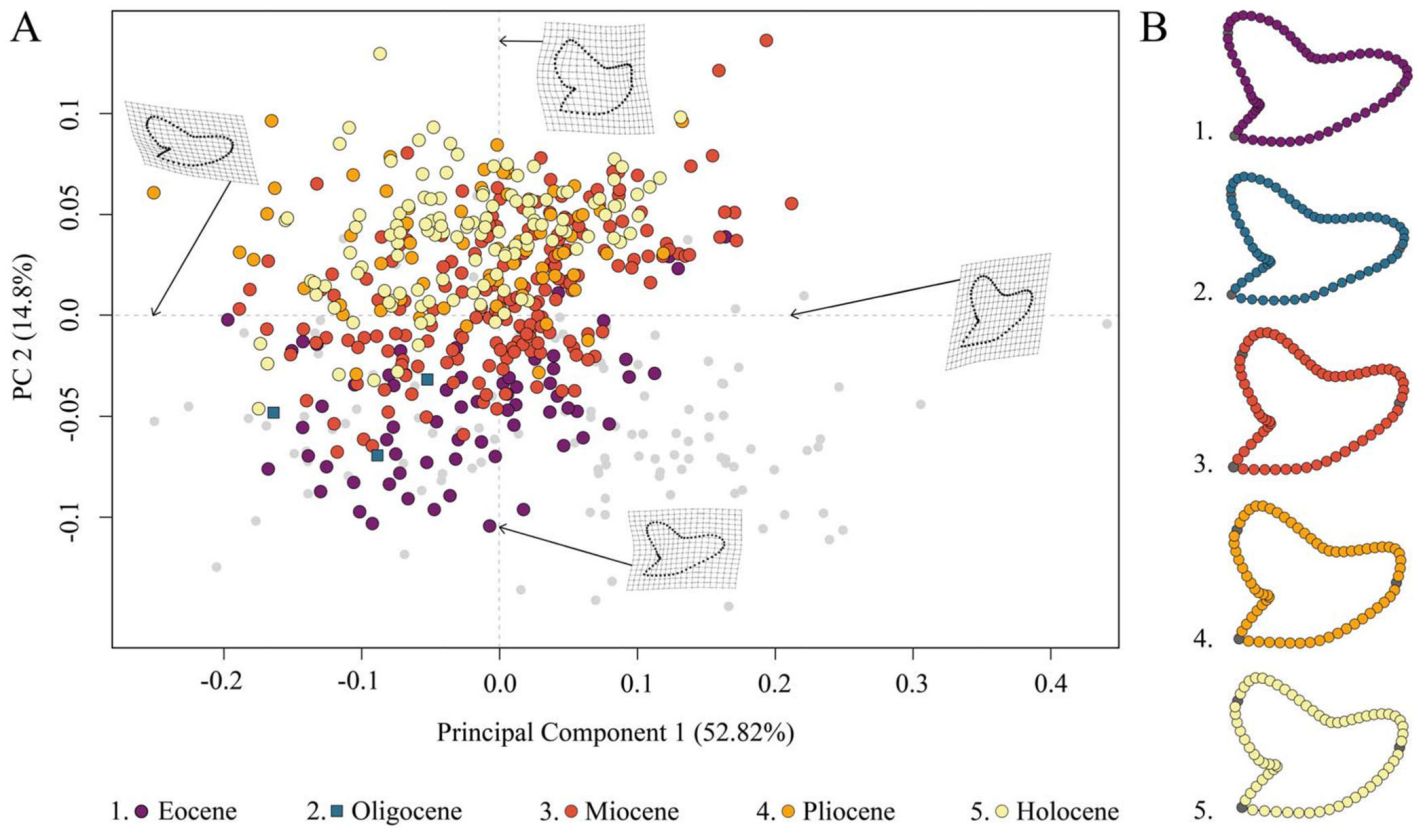
Morphospace occupation of all examined *Galeocerdo* teeth, divided by epochs. A, Scatter plot of the first two principal component (PC) axes. B, Mean tooth shapes of all examined groups.

The species distribution of *Galeocerdo* in the morphospace is related to geologic time and shows a separation of Paleogene from Neogene and younger species along PC 2; Eocene and Oligocene species mainly accumulate in the negative realm, and Miocene species are partly overlapping with both but are better represented by positive values of PC 2. Pliocene and Holocene species, however, are highly overlapping with each other and almost completely separated from Eocene and Oligocene species (Fig. 4).

PC 1 describes the difference between lateral and anterior teeth, with negative scores indicating broad and compressed teeth with a flattened root and a strongly distally inclined primary cusp. Conversely, positive scores are related to high, upright teeth with a V-shaped and broad root. PC 2 explains the morphological change of teeth from Eocene to younger stratigraphic ages, with negative values indicating teeth having a symmetrical root, a slightly curved mesial cutting edge, an obtuse-angled distal notch, and a straight distal heel. Positive scores are related to broad teeth with strongly curved mesial and distal cutting edges, a bulky crown, and a deep distal notch (Fig. 4).

The implemented PERMANOVA suggests that the examined time-bin compositions are significantly different (Table 1), except for the Eocene/Oligocene association and the Pliocene/Holocene association (Table 2). However, only three specimens from the Oligocene could be included in this study, and therefore no statistically relevant conclusions can be reached here.

**Figure 5:**
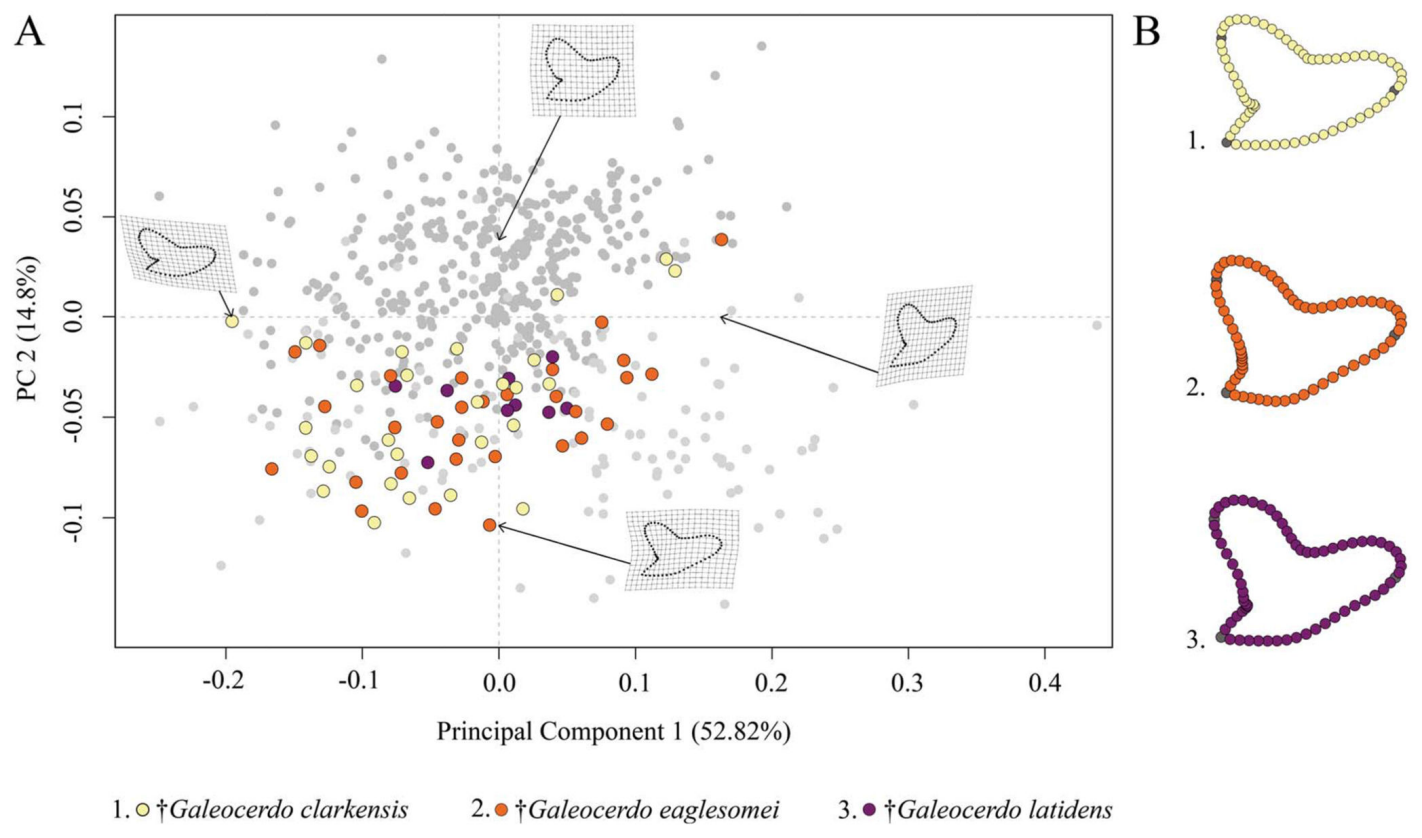
Morphospace of all examined Paleogene *Galeocerdo* teeth. A, Scatter plot of the first two principal component (PC) axes. B, Mean tooth shapes of all examined groups.

*Paleogene Tiger Sharks.—*The Paleogene species †*G*. *clarkensis*, †*G*. *eaglesomei*, and †*G*. *latidens* mainly occupy the negative region of PC 1 and PC 2, with †*G*. *clarkensis* and †*G*. *eaglesomei* being distributed broadly across the shared realm and †*G*. *latidens* accumulating in the center (Fig. 5).

Despite the overlap of the species in the morphospace, a PERMANOVA suggests significant differences between them (Table 1). The pairwise comparison between the species, however, reveals a high degree of overlap between †*G*. *eaglesomei* and †*G*. *latidens*, indicating no significant differences between these two species (Table 2).

#### Neogene–Quaternary Tiger Sharks

**Figure 6:**
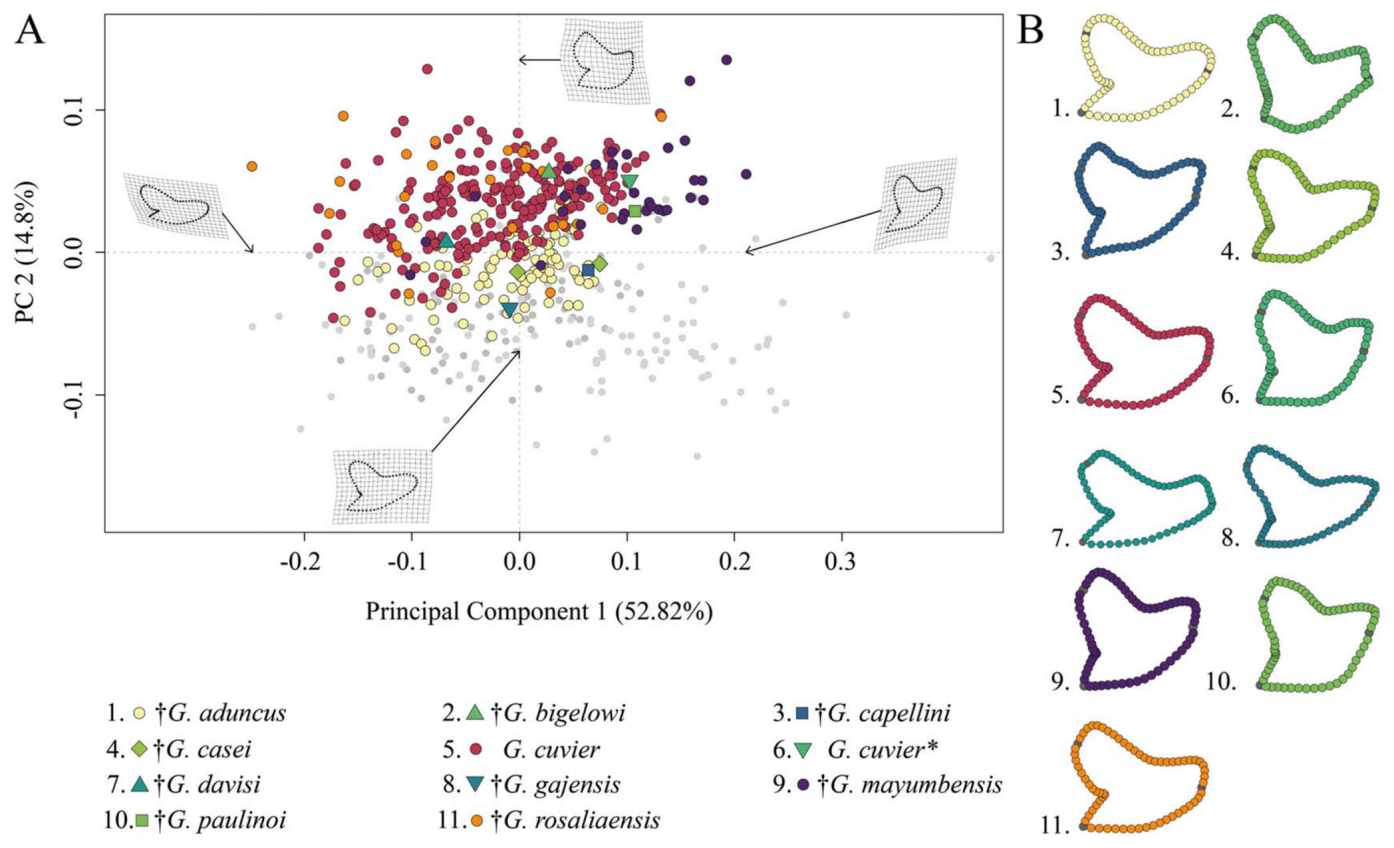
Morphospace of all examined Neogene-Quaternary *Galeocerdo* teeth. A, Scatter plot of the first two principal component (PC) axes. The single tooth described by dos Reis (2005) as *G*. *cuvier* is highlighted with an asterisk. B, Mean tooth shapes of all examined groups.

The morphospace occupation of Neogene–Quaternary (Miocene–Holocene) species shows a broad distribution of *G*. *cuvier* and †*G*. *rosaliaensis*, predominantly in the positive realm of PC 2. †*Galeocerdo aduncus* is partly overlapping with both species in the morphospace but is distributed mainly along the negative scores of PC 2. †*Galeocerdo mayumbensis* is most separated from the others and occupies the positive end of PC 1. Six doubtful species (†*G*. *bigelowi*, †*G*. *casei*, †*G*. *capellini*, †*G*. *davisi*, †*G*. *gajensis*, †*G*. *paulinoi*) were additionally included in the morphospace to determine their species affiliations. Accordingly, †*G*. *capellini* and †*G*. *gajensis*, which are only represented by one tooth each, plot within the morphospace of †*G*. *aduncus*. The single tooth of †*G*. *davisi* is located within the overlapping morphospaces of †*G*. *aduncus*, *G*. *cuvier*, and †*G*. *rosaliaensis*. The highly similar tooth morphology of †*G*. *bigelowi*, †*G*. *casei*, and †*G*. *paulinoi* to that of †*G*. *mayumbensis* is reflected in the morphospace occupation, with †*G*. *bigelowi* and †*G*. *paulinoi* plotting within and †*G*. *casei* plotting extremely close to the morphospace occupied by †*G*. *mayumbensis* (Fig. 6).

The results of the PERMANOVA indicate a significant difference between the examined species (Table 1). This is further corroborated by a pairwise comparison, in which all species are significantly different from one another (Table 2).

#### Dental Disparity through Time

**Figure 7:**
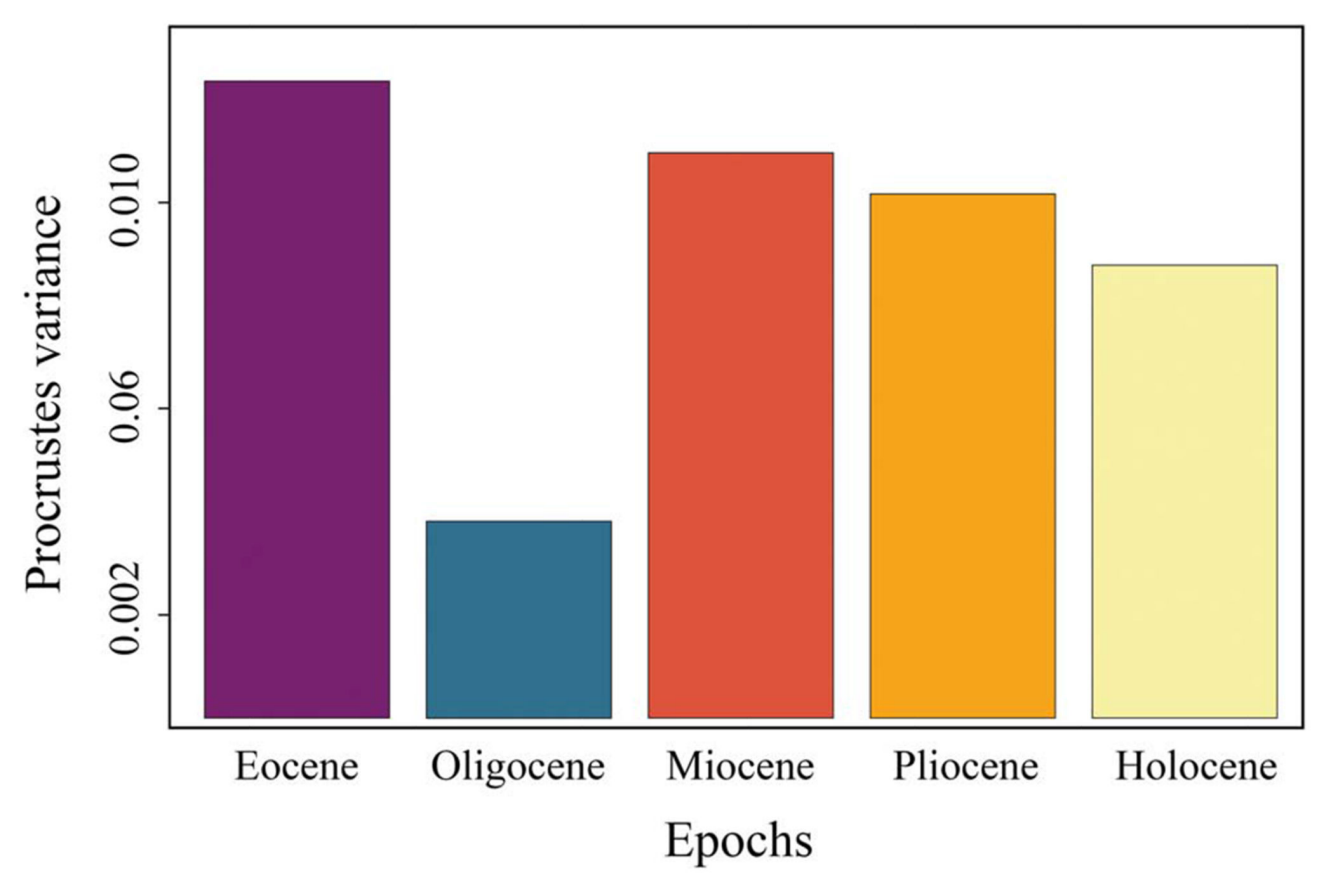
Disparity through time of the dental morphology of *Galeocerdo*.

The dental morphological disparity of *Galeocerdo* attains its maximum in the Eocene, followed by a massive drop, resulting in the lowest disparity level in the Oligocene. During the Miocene, disparity increases again, albeit not to the levels attained in the Eocene, and eventually keeps a high level through the remaining Neogene and Quaternary (Fig. 7, Table 3).

## Discussion

### Geometric Morphometrics

Up to now, more than 60 extinct tiger shark species based on isolated teeth have been described since the first taxonomic description of the extant species ca. 200 years ago. After various revisions, 23 species currently are considered valid, although many remain dubious. To overcome the problem of qualitative morphological characters that often bear the problem of undetected convergences, we additionally used quantitative geometric morphometric analyses here. Unfortunately, it was not possible to obtain teeth, or at least useful figures, of all 23 species, because six species were described without illustration (i.e., †*G*. *aeltrensis*, †*G*. *priscus*, †*G*. *pygmaeus*, †*G*. *similis*, †*G*. *subcrenatus*, and †*G*. *sublaevis*), and figures of †*G*. *productus* were only available in lingual view and we used solely teeth photographed in labial view in the present study to minimize inaccuracies. Nevertheless, we were able to analyze teeth of 16 nominal species (see “Material and Methods” section for detailed account) and compared them with morphologically similar teeth of *Hemipristis*, †*Physogaleus contortus*, and †*P*. *alabamensis*. Our results clearly show that teeth of *Hemipristis*, †*Physogaleus*, and *Galeocerdo* species are well separated despite dental resemblances, especially between *Hemipristis* and †*G*. *eaglesomei*. It is furthermore evident that two species traditionally included in *Galeocerdo*, †*G*. *acutus* and †*G*. *triqueter*, must be included in †*P*. *contortus*, although this should be treated with caution because of the low number of specimens, qualitative characters (e.g., slender and smooth/ minor serrated tooth crowns) further corroborate this result. Unfortunately, no tooth of †*G*. *productus* was available for the quantitative analysis. The characters described by Agassiz (1856), such as only a slightly curved cusp and small serrations at the basis, however, are very similar to dental characters of †*P*. *contortus*. Available illustrations furthermore support the assumption that †*G*. *productus* also is a junior synonym of †*P*. *contortus*.

### Paleogene Tiger Sharks

Three species were not included in this analysis: one “interesting tooth” from the Eocene was described as a new species, †*G*. *aeltrensis* by Van Beneden (1873: p. 385), but without illustrations or further information. Consequently, as descriptions of defining characters as well as illustrations are missing, we consider this species a *nomen nudum*. The second species, †*G*. *priscus*, is based solely upon some isolated scales from the late Eocene (Heckel 1853). Although isolated scales may bear paleoecological signals, they only provide very limited taxonomic information (Ferrón et al. 2014), because different morphotypes occur across the body (e.g., Ankhelyi et al. 2018; Jambura and Kriwet 2020). Due to a lack of an illustration, it is not possible to unambiguously assign the described scales to a species, hence we consider †*G*. *priscus* a *nomen dubium*.

The third species, †*G*. *aegyptiacus*, was described by Stromer (1905) based upon teeth previously assigned to †*G*. *latidens* by Dames (1883) and Stromer (1903). Only very few records of †*G*. *aegyptiacus* are known (e.g., Stromer 1905; Underwood et al. 2011; Malyshkina et al. 2013), and even these are considered doubtful (see Underwood et al. 2011; Sweydan et al. 2019). However, all specimens exhibit a cusp with a smooth distal cutting edge and a mesial cutting edge that is serrated along the lower two-thirds but smooth along the upper third, a character typically observed in the tiger shark–like genus †*Physogaleus* (Ebersole et al. 2019). Recently, Ebersole et al. (2019) erected †*P*. *alabamensis* comb. nov. and included teeth formerly described as *Galeocerdo*, with the same tooth characteristics (e.g., mesial cutting edge serrated on the basis and smooth toward the apex, smooth distal cutting edge, slender and distally directed cusp, coarse serrations on distal heel diminishing in size basally) as seen in the few recorded teeth of †*G*. *aegyptiacus*; hence we propose to combine both species.

We analyzed teeth of the remaining Paleogene species †*G*. *clarkensis*, †*G*. *eaglesomei*, and the putative non-valid †*G*. *latidens*. The original description of †*G*. *eaglesomei* is based on the assumption of Dartevelle and Casier (1943) that teeth figured as †*G*. *latidens* by White (1926) show distinct differences to the holotype of †*G*. *latidens* as described by Agassiz (1843). Nevertheless, Ebersole et al. (2019) synonymized †*G*. *latidens* with †*G*. *eaglesomei*, arguing that both constitute the same species, displaying a certain degree of monognathic heterodonty (i.e., morphological differences between anterior and lateral teeth), as also observed in the extant species, *G*. *cuvier*. Even though †*G*. *latidens* was described before †*G*. *eaglesomei*, Ebersole et al. (2019) decided to use the name †*G*. *eaglesomei* after merging both species, because the locality and horizon of the †*G*. *latidens* holotype are unknown, and therefore, it was considered a *nomen dubium*. Our results clearly support the merging of †*G*. *eaglesomei* and †*G*. *latidens*, as we could not detect significant differences between both species, which is further corroborated by a qualitative comparison of the tooth morphologies.

It is, however, noteworthy that †*G*. *latidens* appears to represent a wastebasket taxon for Eocene teeth over time; teeth originally described as †*G*. *latidens* have been assigned to other *Galeocerdo* species as well as to other genera, including *Carcharhinus* and †*Physogaleus* (see, e.g., Stromer 1905; White 1955; Ebersole et al. 2019). It therefore cannot be ruled out that “†*G*. *latidens”* includes more possibly unrecognized species.

†*Galeocerdo clarkensis* is an Eocene tiger shark species whose teeth differ from those of †*G*. *eaglesomei* in various aspects: the mesial cutting edge is evenly convex rather than sigmoidal (typical for anterior teeth of †*G*. *eaglesomei*), the serrations are compound (presence of secondary serrations on serrae) instead of being simple (Ebersole et al. 2019), and the distal notch is more distinctly developed. This discrimination is also well supported by our results, where †*G*. *clarkensis* is well differentiated from †*G*. *eaglesomei* according to the pairwise comparison, emphasizing the validity of both species.

#### Neogene–Quaternary Tiger Sharks

The Neogene–Quaternary period (Miocene to Holocene) was initially characterized by a high diversity of tiger shark species. Of all species, three could not be included in the analysis: †*G*. *similis*, †*G*. *sublaevis*, and †*G*. *pygmaeus* from the Miocene were only mentioned but neither described nor illustrated (see Münster 1842), making these species *nomina nuda*. Furthermore, our results indicate that †*G*. *triqueter* is a junior synonym of †*P*. *contortus* based on similar tooth morphologies and the same morphospace occupation (see “PCA on the Whole Sample”).

Within the analysis of the remaining Neogene–Quaternary tiger shark species, the most separated and distinct morphospace is the one occupied by †*G*. *mayumbensis*.

The Miocene species †*G*. *bigelowi*, †*G*. *paulinoi*, and †*G*. *casei* already were considered synonymous with †*G*. *mayumbensis* because of qualitative morphological similarities (Andrianavalona et al. 2015; Carrillo-Briceño et al. 2019). In our study, †*G*. *bigelowi* and †*G*. *paulinoi* are both represented by one tooth each, and †*G*. *casei* by only two teeth. Hence, we were not able to provide robust statistical support for this assignment; however, all three species clearly share the same morphospace with †*G*. *mayumbensis*. The bulky tooth crowns, the strongly curved mesial cutting edges, and the Miocene age of all three species furthermore support synonymizing them with †*G*. *mayumbensis*.

In the current study, the Pliocene species †*G*. *capellini* is represented only by one tooth, and consequently, we cannot provide statistically informative results regarding this species. Teeth of †*G*. *capellini* are characterized by a large size and a distinct secondary serration (Lawley 1876). Purdy et al. (2001) suggested merging †*G*. *rosaliaensis* with †*G*. *capellini* because of their similar morphology. Here, the examined teeth of both species plot within the same morphospace, supporting this merger. However, they are also largely overlapping with the extant *G*. *cuvier* and with †*G*. *aduncus*, which occurred until the late Miocene, possibly the early Pliocene. An assignment of both species to †*G*. *aduncus* can be ruled out, because teeth of †*G*. *capellini* and †*G*. *rosaliaensis* possess a distinct secondary serration (as also observed in *G*. *cuvier*) that separates them from the singly serrated †*G*. *aduncus* teeth. An assignment of †*G*. *capellini* and †*G*. *rosaliaensis* to *G*. *cuvier* was discussed before: Lawley (1876) and Applegate (1978) both emphasized the similarity of teeth of †*G*. *capellini* and †*G*. *rosaliaensis*, respectively, to the extant *G*. *cuvier*, and Cigala-Fulgosi and Mori (1979) eventually transferred †*G*. *capellini* to *G*. *cuvier*. However, Purdy et al. (2001) proposed that a final decision must await a study on the dental variation of *G*. *cuvier*. In the present study, the morphospaces of †*G*. *rosaliaensis* and *G*. *cuvier* are highly overlapping, but nevertheless, both groups are statistically different. We therefore follow Purdy et al. (2001) in considering †*G*. *rosaliaensis* a junior synonym of †*G*. *capellini* and keeping it separated from the extant tiger shark *G*. *cuvier*. However, we urge that a detailed reexamination of the holo- and syntypes of †*G*. *capellini* should be undertaken to clarify diagnostic characters for this species, because those described by Lawley (1876) and Applegate (1978) are not exclusive for †*G*. *capellini*. When photographing the specimens described by Applegate (1978) for this study, one of the authors (F.A.L.-R.) noticed an extremely thick root compared with those of the extant *G*. *cuvier*, which could represent a useful character in species discrimination, pending future studies.

Due to the morphological similarity, Purdy et al. (2001) suggested that teeth of †*G*. *aduncus* represent juvenile teeth of the extant *G*. *cuvier*. Traditionally, †*G*. *aduncus* is separated from *G*. *cuvier* based on the size as well as the absence of secondary serrations on the mesial cutting edge (Stoutamire 1975; Applegate 1978; Cigala-Fulgosi and Mori 1979; Kent 2018). The present geometric morphometric analysis depicts a strong overlap of †*G*. *aduncus* and *G*. *cuvier* teeth, especially along the first PC axis, which describes the monognathic heterodonty. However, both species are perceivably more separated along PC 2 (describing shape differences of the root lobe and the breadth of the crown) and are clearly different species according to the ANOVA. These results, in addition to the absence of a secondarily serrated mesial cutting edge, clearly demonstrate that †*G*. *aduncus* has to be considered a valid species.

A secondary serration is also lacking in teeth of †*G*. *davisi* and †*G*. *gajensis*, and furthermore, both species plot within the three overlapping morphospaces of †*G*. *aduncus*, *G*. *cuvier*, and †*G*. *rosaliaensis*. Although both taxa are represented by only one tooth each, which complicates the interpretation of the results, as no statistical clarity is present, the similar tooth morphology and the simple serration clearly indicate that †*G*. *davisi* and †*G*. *gajensis* should be considered synonymous with †*G*. *aduncus*.

A distinct dignathic heterodonty (i.e., morphological differences between teeth of upper and lower jaws) is known from several carcharhiniform sharks (Compagno 1988), with the extant tiger shark *G*. *cuvier* being very exceptional, as the teeth of both jaws are very similar in appearance. A lack of an articulated dentition of any fossil tiger shark species complicates the question of whether extinct *Galeocerdo* species already possessed a monognathic dentition or not. Notably, the dentition of †*G*. *aduncus* was at the center of intensive discussions regarding possible heterodonties for a long time, and different authors provided various hypotheses, including that a well-developed dignathic heterodonty occurred in †*G*. *aduncus*, with †*P*. *contortus*–type teeth in the lower jaw and typical cockscomb-shaped teeth in the upper jaw (Applegate 1978, 1992), or that †*G*. *aduncus* displays a gynandric heterodonty, with only males having slender tooth crowns (Ward and Bonavia 2001). Ward and Bonavia (2001) accordingly assumed that teeth of †*P*. *contortus* represent teeth of male †*G*. *aduncus* and merged both species into †*P*. *aduncus*.

The main reason for rejecting the hypothesis of a distinct dignathic heterodonty with †*G*. *aduncus*–type teeth in the upper jaw and †*P*. *contortus*–type teeth in the lower jaw of †*G*. *aduncus* is the fact that there are localities where only teeth with either one of these two morphologies occur (Kent 2018). A possible gynandric heterodonty, however, would indicate a strong segregation by sex at the respective localities (Reinecke et al. 2011). In the extant tiger shark, sexual separation is only known to occur seasonally at some sites, for example, Tiger Beach in the western central Atlantic (Bahamas), where mostly female sharks of different life stages reside to reach maturity with less male mating harassment and to use the warm environment to reduce the gestation periods (Lea et al. 2015; Sulikowski et al. 2016).

**Figure 8:**
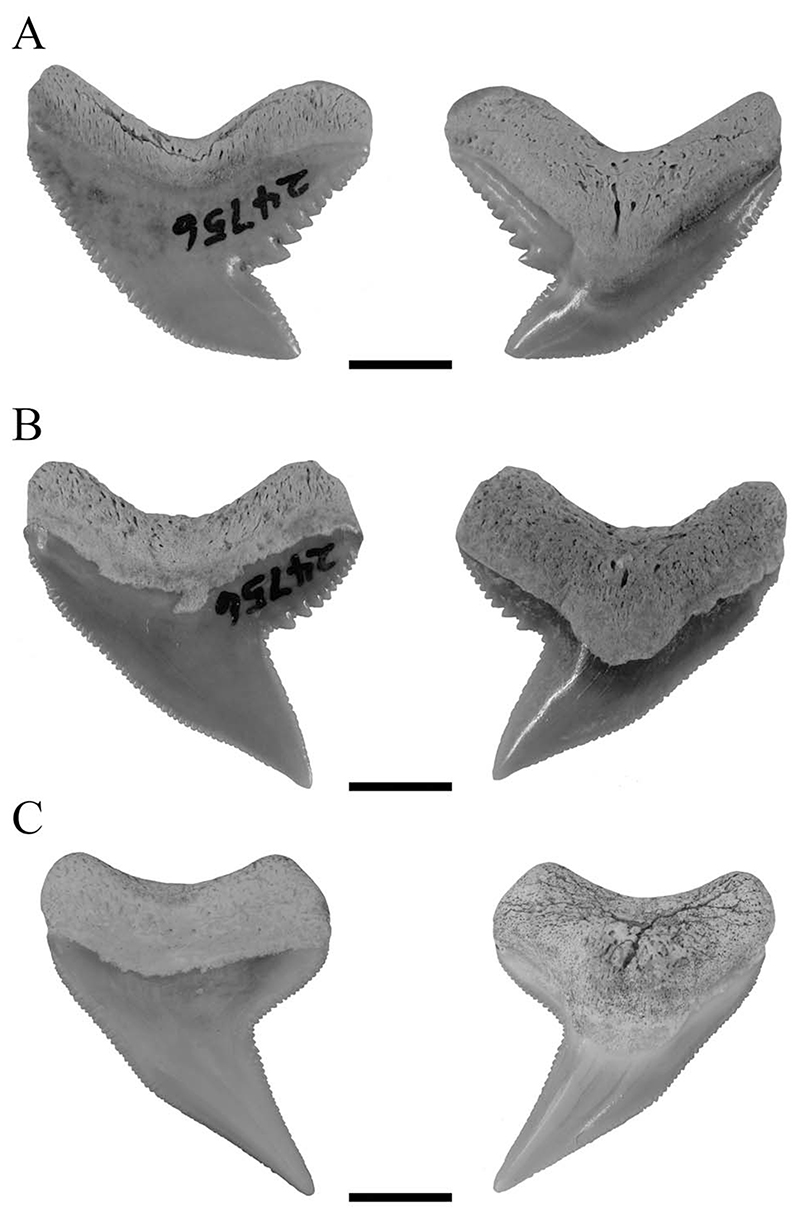
Isolated fossil teeth of †*G*. *aduncus* and †*P*. *contortus* in labial and lingual positions. A, Broad tooth morphology of †*G*. *aduncus*. B, Narrow tooth morphology of †*G*. *aduncus*. C, Typical tooth morphology of †*P*. *contortus*. Scale bars, 5 mm.

Kent (2018) supported the hypothesis of †*G*. *aduncus* and †*P*. *contortus* being separate species but nevertheless assumed a certain heterodonty in †*G*. *aduncus*. He described three types of tooth morphologies for both species, comprising a broad and a narrow morphology in †*G*. *aduncus* and teeth with the typical †*P*. *contortus* morphology. The dataset of †*G*. *aduncus* in the present study contained teeth with broad and narrow morphologies, both clustering together. However, both types of teeth together showed distinct differences to teeth unambiguously representing †*P*. *contortus* (Fig. 8). This demonstrates the presence of a heterodont dentition in †*G*. *aduncus*, with broad and narrow teeth, which nevertheless are clearly different from †*P*. *contortus*, and therefore the validity of both taxa. Although a certain heterodonty, either dignathic or gynandric, seemingly exists in †*G*. *aduncus*, it is not to the extent previously proposed (i.e., including †*P*. *contortus*–type teeth). The presence of broad and narrow tooth morphologies in †*G*. *aduncus* is furthermore a character that differentiates its teeth from those of *G*. *cuvier* and hence can be used to distinguish both species.

**Figure 9:**
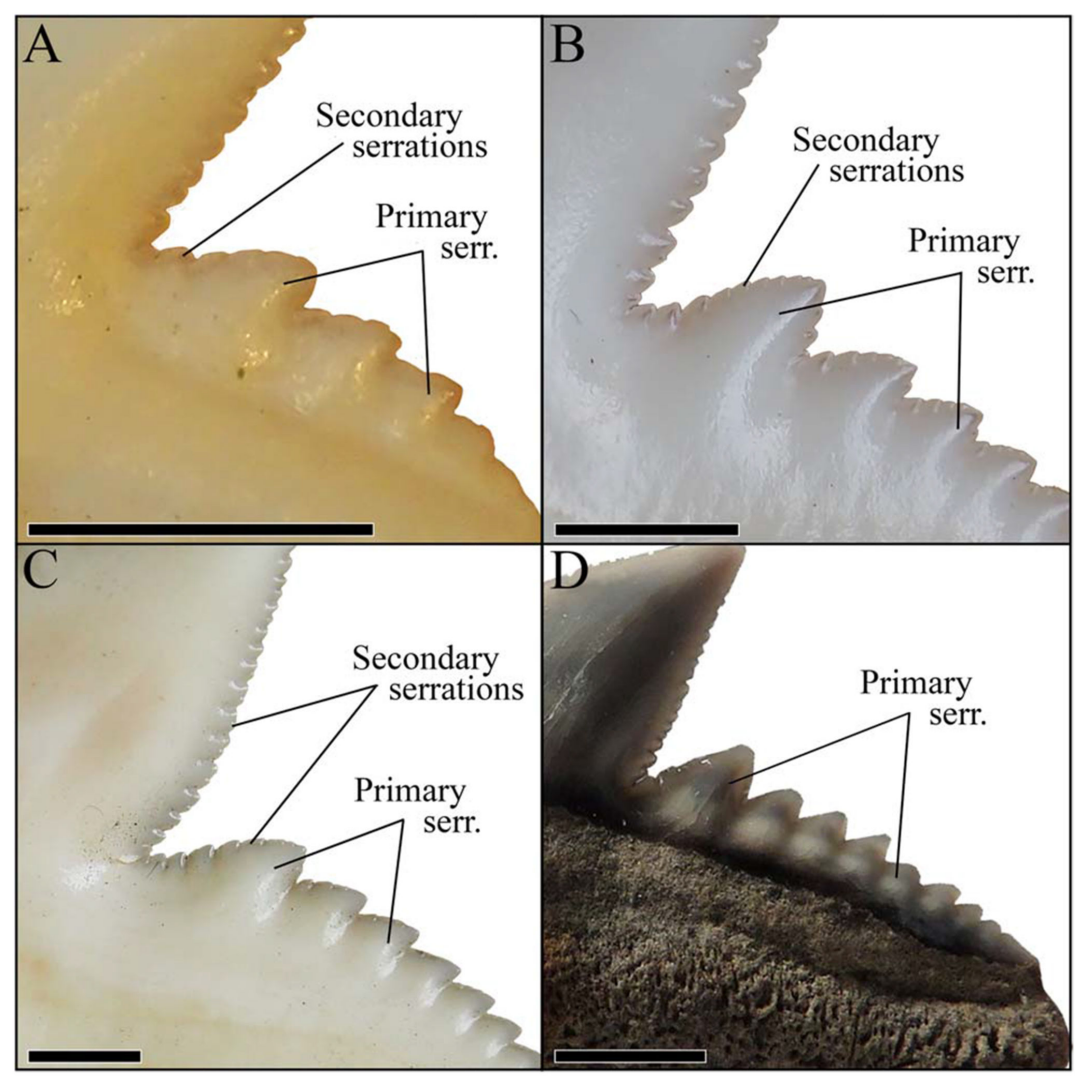
Magnification of the distal cutting edges and distal heels of the teeth of three different *G*. *cuvier* specimens and one †*G*. *aduncus* specimen, identifying the primary and secondary serrations. A, Anterior tooth of a juvenile *G*. *cuvier* specimen. B, Anterior tooth of a subadult *G*. *cuvier* specimen. C, Lateral tooth of an adult *G*. *cuvier* specimen. D, Isolated lateral tooth of †*G*. *aduncus*. Scale bars, 2 mm.

Typically, it is assumed that the sole extant tiger shark *G*. *cuvier* evolved in the late Miocene/early Pliocene. However, dos Reis (2005), Pimiento et al. (2013), and Patnaik et al. (2014) indicated the presence of *G*. *cuvier* in the early Miocene. The tooth described by dos Reis (2005) clearly belongs to †*G*. *mayumbensis*, as demonstrated by our analyses here (Fig. 6), also indicated by Carrillo-Briceño et al. (2019). The teeth described by Pimiento et al. (2013) and Patnaik et al. (2014) as *G*. *cuvier* were not included in this study but already were demonstrated to belong to †*G*. *mayumbensis* by Carrillo-Briceño et al. (2019), an interpretation with which we agree. So far, no unambiguous record of *G*. *cuvier* from the early Miocene has been reported. However, 37 teeth from middle Miocene deposits of Florida, USA (Supplementary Table 2), are identified unambiguously as those of *G*. *cuvier* here, based on distinct diagnostic characters for *G*. *cuvier* teeth, such as the presence of secondary serrations (Fig. 9). These records extend the origin of the extant species from the late Miocene/early Pliocene (ca. 5.3 Ma) back into the middle Miocene (ca. 13.8 Ma).

### Disparity Patterns through Time

When the number of tiger shark species observed in this study is compared to the dental disparity of *Galeocerdo* through time, the most conspicuous feature is the contrasting development of both. Although only two validated taxa of tiger sharks were present throughout the Eocene (†*G*. *clarkensis* and †*G*. *eaglesomei*), the disparity of the dental morphology attained its highest levels during this time. Conversely, four species are present throughout the Neogene to Quaternary (†*G*. *aduncus*, †*G*. *capellini*, *G*. *cuvier*, †*G*. *mayumbensis*), but the dental disparity was lower than in the Eocene. Another striking feature is the extremely high disparity from the middle Miocene to today, although the species diversity decreased even further to a single extant species. However, patterns with the highest disparity attained early in the evolution were likewise observed for several other clades (e.g., Erwin 2007; Hughes et al. 2013).

In contrast to †*G*. *eaglesomei*, †*G*. *clarkensis* already developed the typical cockscombshaped dentition, resembling that of the extant *G*. *cuvier*. Hence, the teeth of †*G*. *eaglesomei*, characterized by the weakly developed distal notch distinguishing it from all other tiger shark species, are especially accountable for the high disparity in the Eocene. The Eocene/Oligocene transition was a period of substantial extinction and global ecological change due to decreasing sea temperatures and ice sheet formation on Antarctica (e.g., Coxall and Pearson 2007; Goldner et al. 2014). During this time, the dental disparity of tiger sharks dropped to its lowest level, congruent with the extinction of †*G*. *clarkensis* and †*G*. *eaglesomei* and the origination of †*G*. *aduncus*. However, the low number of Oligocene specimens included in this study (*n* = 3) does not allow statistically significant conclusions, and the possibility of a higher dental disparity of tiger sharks cannot be excluded. Throughout the Neogene and Quaternary, the diversity of tiger sharks increased to four species (†*G*. *aduncus*, †*G*. *capellini*, *G*. *cuvier*, †*G*. *mayumbensis*). Both †*G*. *aduncus* and †*G*. *mayumbensis* vanished in the late Miocene/early Pliocene, and †*G*. *capellini* is only known from Pliocene deposits so far, resulting in only a single extant species, *G*. *cuvier*. Instead of dropping considerably with the taxonomic diversity, the dental disparity decreased only sparsely and remained at a high level from the middle Miocene until today, a pattern attributed to the highly similar tooth morphologies of the Neogene–Quaternary species, especially of †*G*. *aduncus*, †*G*. *capellini*, and *G*. *cuvier*. A similar pattern was observed across the Cretaceous/Paleogene mass extinction: although 84% of all shark species were lost during this event (Kriwet and Benton 2004), the dental disparity of lamniform and carcharhiniform sharks stayed nearly static (Bazzi et al. 2018).

## Conclusions

**Figure 10:**
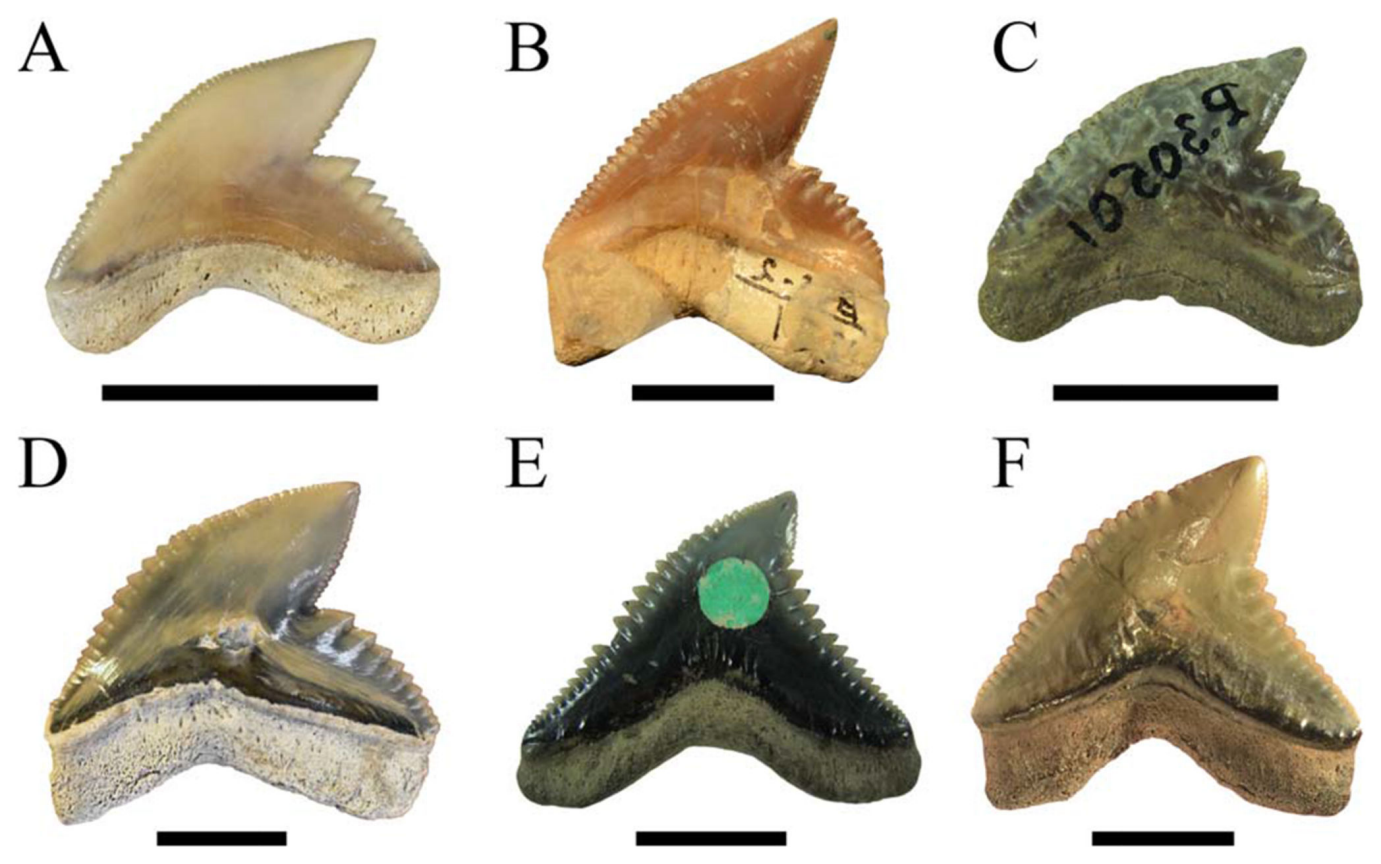
Isolated fossil teeth of the six valid tiger shark species. A, †*Galeocerdo aduncus*. B, †*Galeocerdo capellini*. C, †*Galeocerdo clarkensis* holotype. D, *Galeocerdo cuvier*, E, †*Galeocerdo eaglesomei* holotype. F, †*Galeocerdo mayumbensis*. Scale bars, 10 mm.

We demonstrate here that the presumed high taxonomic diversity of extinct tiger sharks, compared with only one extant species, was much lower than generally assumed. Applying multivariate analyses, we were able to successfully distinguish between six tiger shark species from the Eocene to Holocene instead of 23 (Figs. 1, 10). These species comprise the Eocene †*G*. *clarkensis* and †*G*. *eaglesomei*, the Oligocene to late Miocene †*G*. *aduncus*, the Miocene †*G*. *mayumbensis*, the Pliocene †*G*. *capellini*, and the extant tiger shark *G*. *cuvier*, with a range extension back into the middle Miocene. Our results corroborate that the combined approach of quantitative geometric morphometric techniques and qualitative morphological comparisons is appropriate to identify taxa known only by isolated teeth and to differentiate between teeth of extinct and extant species despite high morphological resemblances. The new analyses moreover show that declining diversity and disparity are not implicitly correlated, which is revealed by the high dental disparity of *Galeocerdo* since the Miocene despite the decrease from four to only one species. Further studies on the dental disparity in sharks in deep time are mandatory to enhance our understanding of extinction patterns and the inherent link between taxonomic diversity and morphological disparity in this group of marine apex predators.

## Supplementary Material

Supplementary data

## Figures and Tables

**Table 1:** Results of the permutational analysis of variance to test for differences in tooth shape between the examined groups. An asterisk indicates a p-value < 0.05. SS, sum of squares; MS, mean squares.

Group	Effect	df	SS	MS	*R^2^*	*F*	*p*
Total sample	Genus	2	1.1479	0.57393	0.1237	39.948	0.001*
	Residuals	566	8.1317	0.01437	0.8763		
	Total	568	9.2796				
*Galeocerdo* only	Epoch	4	0.7280	0.181997	0.1365	17.31	0.001*
	Residuals	438	4.6052	0.010514	0.8635		
	Total	442	5.3332				
*Galeocerdo* only	Species	6	1.3366	0.222770	0.25062	24.302	0.001*
	Residuals	436	3.9966	0.009167	0.74938		
	Total	442	5.3332				

**Table 2:** Results of the pairwise comparison to test for differences in tooth shape between the examined groups. Signficance is depicted as p-value (an asterisk indicates a p-value < 0.05). d, distance; UCL, upper confidence limit; Z, Z-score.

		d	UCL (95%)	Z	^*p*^
Genera	*Galeocerdo*: *Hemipristis*	0.22105688	0.06072138	13.18929	0.001*
	*Galeocerdo*: †*Physogaleus*	0.08449665	0.02238477	13.76419	0.001*
	*Hemipristis*: †*Physogaleus*	0.18821240	0.06107171	11.02890	0.001*
Epochs	Eocene: Oligocene	0.10098920	0.10209586	1.854888	0.055
	Oligocene: Miocene	0.12911354	0.10152548	3.221054	0.007*
	Miocene: Pliocene	0.04564208	0.02440129	5.873084	0.001*
	Pliocene: Holocene	0.02514313	0.02663928	1.632904	0.068
Species	†*G*. *clarkensis:* †*G*. *eaglesomei*	0.07441448	0.04510858	4.942268	0.001*
	†*G*. *clarkensis:* †*G*. *latidens*	0.07202117	0.06429810	2.363137	0.023*
	†*G*. *eaglesomei*: †*G*. *latidens*	0.05405425	0.06630524	1.087175	0.124
	†*G*. *aduncus*: †*G*. *rosaliaensis*	0.07196333	0.03722257	6.309912	0.001*
	†*G*. *aduncus*: *G*. *cuvier*	0.04839204	0.02073741	7.789240	0.001*
	†*G*. *aduncus*: †*G*. *mayumbensis*	0.13711298	0.03392790	14.400205	0.001*
	*G*. *cuvier*: †*G*. *rosaliaensis*	0.03718237	0.03594076	2.130237	0.042*
	*G*. *cuvier*: †*G*. *mayumbensis*	0.13906022	0.03221319	15.260713	0.001*
	†*G*. *mayumbensis*: †*G*. *rosaliaensis*	0.15042923	0.04533909	11.932072	0.001*

**Table 3:** Results of the morphological disparity through time analysis.

Groups	Procrustes variances	
Eocene	0.012359558	
Oligocene	0.003825272	
Miocene	0.010969012	
Pliocene	0.010168379	
Holocene	0.008785643	
	Pairwise distances	^*p*^
Eocene : Oligocene	0.008534286	0.074
Oligocene : Miocene	0.007143740	0.120
Miocene : Pliocene	0.0008006327	0.522
Pliocene : Holocene	0.001382737	0.309

## Data Availability

Data available from the Dryad Digital Repository: https://doi.org/10.5061/dryad.rv15dv47c.
