# Supplementary material for: Evolution, diversity, and disparity of the tiger shark lineage Galeocerdo in deep time
Source: Paleobiology. Author manuscript; Available in PMC 2021 Dec 2. (PMC7612061; doi:10.1017/pab.2021.6)
Supplement: Supplementary data [file EMS135981-supplement-Supplementary_data.zip › Supplementary_Material.pdf]

Evolution, diversity, and disparity of the tiger shark lineage *Galeocerdo* in deep time

Julia Türtscher[1]*, Faviel A. López-Romero[1], Patrick L. Jambura[1], René Kindlimann[2], David J. Ward[3], Jürgen Kriwet[1]

Affiliations

[1]University of Vienna, Department of Palaeontology, Vienna, Austria

[2]Haimuseum und Sammlung R. Kindlimann, Aathal-Seegräben, Switzerland

[3]Natural History Museum, Department of Earth Sciences, London, UK

*Corresponding Author

E-mail: tuertscher.julia@gmail.com

Supplementary Material

Dentition and tooth characteristics of *Galeocerdo cuvier* (Péron & Lesueur, 1822)

The teeth of *Galeocerdo cuvier* are very distinctive and unique as they exhibit a characteristic cockscomb-shape. The crown is compressed and distally inclined. The mesial cutting edge is curved, whereas the distal edge is deeply notched and divided into the distal heel and distal cutting edge (Fig. 1). The crown is completely serrated with compound serrations, whereby large primary serrations are located on the mesial cutting edge and the distal heel and secondary serrations are situated on and between primary serrations as well as on the distal cutting edge (Moyer and Bemis 2017). The asymmetrical, bean-shaped root is compressed and possesses rounded root lobes and a slight lingual protuberance with a nutrient groove.

The teeth of the upper and lower jaws barely differ from each other. Consequently, the dignathic heterodonty is very weak with the upper teeth being slightly larger than lower ones (Snodgrass and Heller 1905; Sarangdhar 1943; Hooijer 1954; Purdy et al. 2001). On the contrary, the monognathic heterodonty is well developed in tiger sharks, as there are vast morphological differences between teeth along the horizontal rows in both jaws. The more posterior the teeth are located, the broader they get and in the end the width exceeds the height of the teeth. A weak ontogenetic heterodonty is present as teeth of juvenile sharks possess fewer serrations and narrower, more elongated cusps (Compagno 1988).

Teeth of tiger sharks display an orthodont histology, with osteodentine confined to the root and a thick layer of orthodentine surrounding a prominent pulp cavity (Applegate 1978; Jambura et al. 2018). The orthodentine of the crown is coated with multilayered enameloid.

Fossil teeth of the extant tiger shark *G. cuvier* are present since the late Miocene/early Pliocene (ca. 5.3 Ma; Cappetta 1987; Müller 1999; Cappetta 2006). Teeth from the early Miocene (ca. 20.4 Ma), reported by Pimiento et al. (2013), dos Reis (2005) and others were re-

assigned to †*Galeocerdo mayumbensis* by Carrillo-Briceño et al. (2019). Carrillo-Briceño et al. (2015) described two *G. cuvier* teeth from middle to late Miocene (ca. 13.8 – 7.2 Ma) formations, however, without going into detail regarding the presence of compound serrations on the mesial cutting edge.

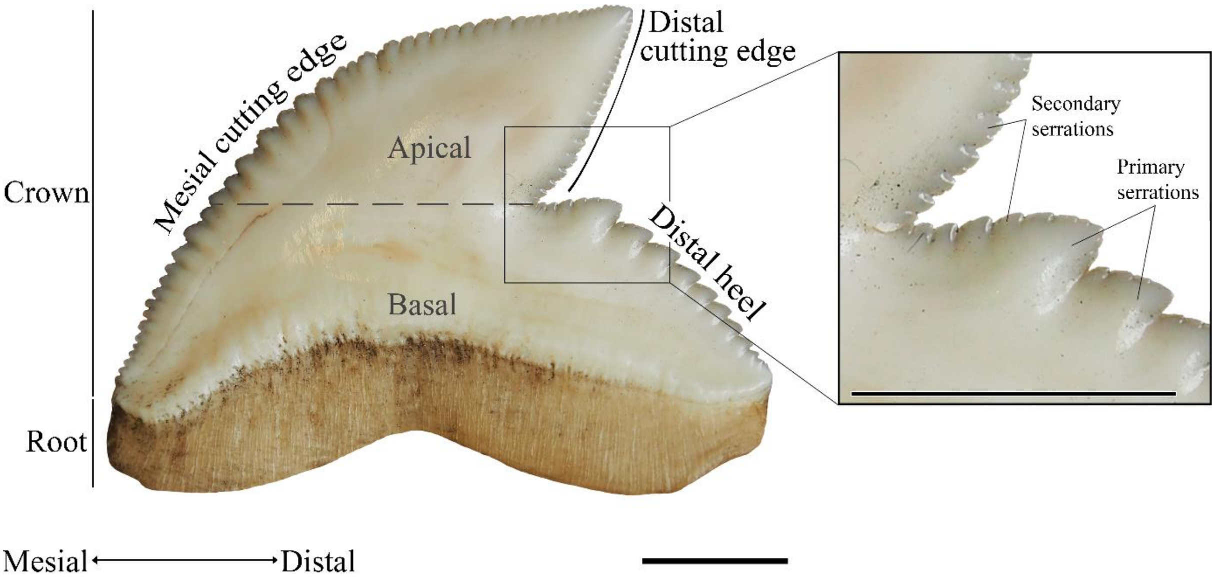

FIGURE 1: Photograph of the labial side of a lateral tooth of an adult *G. cuvier* specimen (EMRG-Chond-J-16), identifying the apical and basal part of the tooth, the crown, root, mesial and distal cutting edges and the distal heel. The magnification shows the primary and secondary serrations. Scale bars, 5 mm.

Fossil record of the genus *Galeocerdo* Müller & Henle, 1837

†*Galeocerdo acutus* Storms, 1894 (re-assigned to †*P. contortus*)

Description – The holotype of †*G. acutus* has a broad and symmetric root and a slender crown. The cusp is very pointy and the mesial cutting edge is slightly curved and possesses small serrations on the basis but not near the apex. Small serrations are present on the distal cutting edge and distal heel as well, with the serrations on the heel being larger and more

marked. Storms (1894) furthermore described the distal cutting edge as being slightly curved (Fig. 2A).

Discussion – †*Galeocerdo acutus* is known from the early Oligocene, Rupelian (ca. 33.9 Ma; Storms 1894; Leriche 1910). The original description of this species is based on a single tooth only (Storms 1894), but Leriche (1910) later reported of a second tooth. Subsequently, based on morphological similarities, it was suggested that †*G. acutus* is a junior synonym of †*G. contortus* (Purdy et al. 2001; Marsili et al. 2007), and †*Physogaleus contortus,* respectively (Reinecke et al. 2011). Due to the similar tooth morphology, we agree with this interpretation to re-assign †*G. acutus* to †*P. contortus*.

†***Galeocerdo aduncus*** Agassiz, 1843 (valid)

Description – The holotype of †*G. aduncus* shows a high degree of similarity to teeth of the extant *G. cuvier*: the root is moderately thick and slightly asymmetric, the crown is compact with the serrated mesial cutting edge greatly curved. The distal part of the teeth is serrated as well, with small serrations on the cutting edge and larger ones on the heel (Fig. 2B).

Discussion – Known from Oligocene (ca. 33.9 Ma) to late Miocene (ca. 5.3 Ma) deposits, teeth of †*G. aduncus* highly resemble those of the modern tiger shark *G. cuvier* (Agassiz 1843; Giebel 1848; Purdy et al. 2001). Agassiz (1843) separated †*G. aduncus* from *G. cuvier* on the basis that the distal heel exhibits a greater convexity in the fossil species, a character which is present and variable in the extant species as well (Purdy et al. 2001). Subsequently described diagnostic characters of †*G. aduncus* are the lack of secondary serrations on the mesial cutting edge (Stoutamire 1975; Cigala-Fulgosi and Mori 1979), a thinner, less robust crown (Stoutamire 1975; Applegate 1978) and a smaller overall tooth size than in *G. cuvier* (Applegate 1978; Kent 2018). Applegate (1978, 1992) stated that †*G. aduncus* exhibits a high degree of heterodonty, in contrast to all other tiger shark species. However, it frequently occurs

that teeth of †*P. contortus* are listed as lower teeth of †*G. aduncus* and Ward and Bonavia (2001) merged †*G. aduncus* with †*P. contortus* together into †*Physogaleus aduncus* assuming gynandric heterodonty (only males had lower teeth with †*P. contortus* morphology). On the contrary, Purdy (1998) and Reinecke et al. (2011) rejected this hypothesis, separated the two morphotypes and referred the presumed upper teeth to †*G. aduncus* and the presumed lower teeth to †*Galeocerdo contortus* (Purdy 1998), respectively †*Physogaleus contortus* (Reinecke et al. 2011). Kent (2018), however, distinguished between a broad and narrow tooth morphotype in †*G. aduncus* and differentiated the narrow tooth morphology from that of †*P. contortus* and, like Applegate (1978, 1992) before, suggested a certain heterodonty in †*G. aduncus*, either dignathic or gynandric. Describing the teeth of †*G. aduncus* as intermediate between those of *Galeocerdo* and †*Physogaleus*, Andrianavalona et al. (2015) even proposed to place †*G. aduncus* in a separate genus. Purdy et al. (2001), however, considered †*G. aduncus* a *nomen dubium* since the holotype is lost and potentially represented a juvenile *G. cuvier* specimen. Reinecke et al. (2011) rejected this deliberation, trusting that the holotype will be re-discovered in the future and the validity of †*G. aduncus* will be confirmed. Furthermore, they relied on the common assumption that fossils of *G. cuvier* appear only since the Pliocene and not already in the late Miocene. In the present study, morphological differences (e.g., absence of secondary serrations) and statistically significant differences in the geometric morphometric analysis to the extant *G. cuvier* clearly support the validity of †*G. aduncus*.

†***Galeocerdo aegyptiacus*** Stromer, 1905 (combined with †*P. alabamensis*)

Description – According to the description of Stromer (1905), teeth of †*G. aegyptiacus* are small with a larger width than height. The root is slender and arched, the short cusp is pointed. The mesial cutting edge lacks serrations near the apex, the distal cutting edge is completely smooth. Large serrations are present on the convex distal heel (Fig. 2C).

Discussion – †*Galeocerdo aegyptiacus* is known from the Eocene, Bartonian (ca. 41.2 Ma) to Priabonian (ca. 33.9 Ma; Cappetta 2006; Malyshkina et al. 2013). Stromer (1905) established †*G. aegyptiacus* based on the assumption that teeth described by Dames (1883) as †*G. latidens* constitute a new species. The description lacks a clear and detailed figuration, however, Stromer (1905) noted that another tooth initially described as †*G. latidens* earlier by himself (Stromer 1903) belongs to the new species as well. The depiction of this apparently anterior tooth is considerably better than the one of the holotype and the described characteristics such as a smooth cusp (no serrations on the distal cutting edge and on the mesial cutting edge near the apex) are well discernible. However, the type material seemingly got lost during World War 2 and as there is no clear figuration of it, Sweydan et al. (2019) recently characterized this species as ambiguous without considering the depiction by Stromer (1903).

In the course of their extensive study on fossil shark teeth, Ebersole et al. (2019) considered †*G. latidens* a *nomen dubium* and re-assigned the teeth to different species, including †*G. eaglesomei* and †*Physogaleus alabamensis*. †*Galeocerdo aegyptiacus*, also derived from teeth originally referred to †*G. latidens*, shares considerable characteristics with †*P. alabamensis*, most importantly the smooth cusp, hence we suggest to combine both species.

†***Galeocerdo aeltrensis*** Van Beneden, 1873 (*nomen nudum*)

Discussion – In a paper lacking both an illustration and a detailed description, Van Beneden (1873: p. 385) only mentioned that he was given one “interesting tooth” from the Eocene, Lutetian (ca. 47.8 Ma) of Belgium, Aeltre and named it †*Galeocerdo aeltrensis*. Because of this absence of information, we regard this species a *nomen nudum.*

†***Galeocerdo bigelowi*** (Mehrotra, Mishra & Srivastava, 1973) (re-assigned to †*G. mayumbensis*)

Description – †*Galeocerdo bigelowi* has a very compact tooth morphology, with a thick and V-shaped root and a very broad crown. The mesial cutting edge is highly curved, the distal notch is very obtuse-angled and the distal heel very steep. The crown is fully serrated; a secondary serration is possible but not discernible by the published illustrations due to their low quality (Fig. 2D).

Discussion – Originally described as †*Carcharodon bigelowi*, Cappetta (2006) assigned this species from the Miocene (Aquitanian/Burdigalian, ca. 20.4 Ma) to *Galeocerdo*. Carrillo-Briceño et al. (2019) later assumed that it represents †*G. mayumbensis*, an opinion we agree on due to unambiguous morphological similarities.

†***Galeocerdo capellini*** Lawley, 1876 (valid)

Description – The tooth described by Lawley (1881) is large (3 cm in width, 2.8 cm in height) and has a broad root. The crown is fully serrated, albeit the serrations on the midway of the mesial cutting edge and especially the distal heel are the largest. Lawley (1876, 1881) furthermore described a marked secondary serration (Fig. 2E).

Discussion – About 40 teeth of †*G. capellini* were found in Pliocene (Zanclean, ca. 5.3 Ma) deposits of Italy (Lawley 1876, 1881). Although Lawley (1876) pointed out that the teeth are very similar to those of the extant species, de Stefano (1909) synonymized the species with †*G. aduncus.* However, based on the secondary serrations, the similar morphology and size of the teeth they were transferred to *G. cuvier* by Cigala-Fulgosi and Mori (1979). Purdy et al. (2001), however, suggested to synonymize †*G. rosaliaensis* with †*G. capellini* and to await a decision regarding the possible affiliation to *G. cuvier*. Here, we agree with Purdy et al. (2001) to await

the final assignment of †*G. capellini* because the unique defining characters have to be specified first.

†***Galeocerdo casei*** Müller, 1999 (re-assigned to †*G. mayumbensis*)

Description – The holotype of †*G. casei* is a large tooth with a broad and V-shaped root. Both the mesial and the distal cutting edges of the tooth are entirely serrated, with the serrations diminishing in size towards the apex. The mesial cutting edge is curved in a slight S-shape and the notch that divides the distal side into a heel and cutting edge is obtuse angled. The tooth moreover possesses distinct secondary serrations as described by Müller (1999; Fig. 2F).

Discussion – †*Galeocerdo casei* is a tiger shark species based on four isolated teeth from the early Miocene (Aquitanian, ca. 23 Ma) of North Carolina (Müller 1999). The teeth of this species are nearly as large as teeth of the extant *G. cuvier*, but with clear morphological differences (Müller 1999). According to Müller (1999), they resemble teeth of †*G. latidens*, †*G. eaglesomei*, †*G. clarkensis* and *G. cuvier* and therefore, †*G. casei* represents a species which lies in between these three Eocene species and the modern *G. cuvier*. Müller (1999) suggested furthermore that the *cuvier*-lineage, from which the recent tiger shark arose, developed from †*G. eaglesomei* over †*G. clarkensis* to †*G. casei* to *G. cuvier*, indicating anagenetic evolutionary scenarios. However, we agree with the interpretation of Andrianavalona et al. (2015), who suggested to synonymize †*G. casei* with the Miocene species †*G. mayumbensis* due to clear morphological similarities.

†***Galeocerdo clarkensis*** White, 1956 (valid)

Description – The holotype of †*G. clarkensis* measures about 1.6 cm in width and has a symmetric, bean-shaped root. The crown is compact with a short and broad cusp. The mesial cutting edge is well curved, with serrations at the basis that diminish in size towards the apex.

The apex is not serrated. Small serrations are present on the distal cutting edge, coarse serrations with incipient secondary serrations on the distal heel (Fig. 2G).

Discussion – Teeth of †*G. clarkensis* were recovered from late Eocene deposits (Priabonian, ca. 37.8 Ma; White 1956; Müller 1999; Cappetta 2006) and were described by White (1956) as a mixture of †*G. aduncus* and †*G. alabamensis*. Westgate (1984) reported similarities of the teeth of †*G. alabamensis* and †*G. clarkensis* and subsequently, †*G. clarkensis* was transferred to †*G. alabamensis* (Manning 1990; Breard 1991; Parmley and Cicimurri 2003; Maisch et al. 2014; citing Manning and Standhardt 1986). Ebersole et al. (2019) eventually re-examined the holotype of †*G. alabamensis,* recognized distinct characteristics to even re-assign this species to the genus †*Physogaleus* and established the species †*P. alabamensis*. Distinct differences between both species, such as secondary serrations on the teeth of †*G. clarkensis* and simple serrations on those of †*P. alabamensis,* clearly support that both species are separated. The present study furthermore indicates the validity of †*G. clarkensis.*

†***Galeocerdo davisi*** Chapman & Pritchard, 1904 (re-assigned to †*G. aduncus*)

Description – The holotype of †*G. davisi* has a broad root and a slender, depressed and pointed crown with a slightly curved mesial cutting edge. The serrations on the mesial cutting edge are very minor, the ones on the distal heel are more marked (Fig. 2H).

Discussion – Davis (1888) originally described this species from the middle Miocene (Langhian, ca. 15.9 Ma) of New Zealand as †*Notidanus marginalis* but included a tiger shark tooth in his description (Chapman and Pritchard 1904). Subsequently, Woodward (1889) suggested to refer this presumed upper tooth of †*N. marginalis* to the genus *Galeocerdo* and to retain the specific name *marginalis* for the newly described *Notidanus* (valid: *Hexanchus*) species. Therefore, Chapman and Pritchard (1904) established the new species †*Galeocerdo davisi* based on this single tooth and pointed out that it is similar to teeth of †*G. latidens* and

†*G. aduncus*. However, shared morphological characters and a huge similarity to teeth of †*G. aduncus* lead us in the present study to the re-assignment of †*G. davisi* to †*G. aduncus*.

†***Galeocerdo eaglesomei*** White, 1955 (valid)

Description – The tooth described by White (1955) is large, has a heavy base with a thick and arched root and a broad crown. The distal cutting edge is short and the obtuse-angled notch is only little developed. The serrations on the mesial cutting edge and distal heel are very coarse and marked and no secondary serrations are present (Fig. 2I).

Due to the weakly formed distal notch in teeth of †*G. eaglesomei* compared to other *Galeocerdo* species, teeth of this species may be confused with other carcharhinid sharks. Samonds et al. (2019), e.g., spuriously identified teeth of †*Carcharhinus balochensis* as being †*G. eaglesomei* and extended the range of the latter to the early Oligocene. Although very similar, there are clear differences between teeth of †*C. balochensis* and †*G. eaglesomei*: the upper anterior teeth of †*C. balochensis* have no marked notch and therefore also lack a distal heel, furthermore both the mesial and distal cutting edges are secondarily serrated. Teeth of †*G. eaglesomei*, however, although not as well marked as in other *Galeocerdo* species, exhibit a distal notch, which divides the distal part of the crown into a cutting edge and a heel. Also, a secondarily serrated cutting edge is not present in †*G. eaglesomei*, which is characterized by possessing coarse primary serrations only. Samonds et al. (2019) discussed the lack of a secondary serration in †*G. eaglesomei* but nevertheless re-assigned †*C. balochensis* to the former taxon, an opinion which is rejected here.

Discussion – Teeth of †*G. eaglesomei* have been reported from middle (ca. 41 Ma) to latest Eocene sediments (ca. 33.9 Ma; Sweydan et al. 2019). They were originally referred to †*G. latidens* (Westgate 1989, citing White 1926). However, Dartevelle and Casier (1943) pointed out some distinct differences between these teeth and those of †*G. latidens*. White (1955)

agreed with this interpretation and subsequently established the species †*G. eaglesomei* due to these differences and also re-assigned the teeth described as †*G. latidens* by Stromer (1905) to this new species. Ebersole et al. (2019), however, synonymized †*G. latidens* with †*G. eaglesomei*, stating that both arguably constitute the same species and that White (1955) simply did not recognize the degree of monognathic heterodonty. The present study clearly confirms this interpretation of †*G. latidens* teeth constituting lateral teeth of †*G. eaglesomei* by employing geometric morphometric techniques, hence we support the merger of both species.

†***Galeocerdo gajensis*** Tewari, Chaturvedi & Singh, 1960 (re-assigned to †*G. aduncus*)

Description – The basis of the holotype of †*G. gajensis* is broad, the curved mesial cutting edge and distal heel are coarsely serrated, the distal cutting edge conversely only slightly. No secondary serrations are present on the broad crown (Fig. 2J).

Discussion – The description of †*G. gajensis* is based on a single tooth from the Miocene (Burdigalian, ca. 18.2 Ma) of India (Tewari et al. 1960; Cappetta 2006). Tewari et al. (1960) pointed out that the tooth resembles teeth of both †*G. aduncus* and *G. rayneri*. Interestingly, they described it as very distinct from *G. tigrinus* (Tewari et al. 1960), which is, as we know today, besides *G. rayneri* and some others, a junior synonym of *G. cuvier*. However, according to Cappetta (2006), †*G. gajensis* might be a junior synonym of †*G. aduncus*. The overall tooth morphology and characters like the absence of a secondary serration support this interpretation by Cappetta (2006), hence we assign †*G. gajensis* to †*G. aduncus* here.

†***Galeocerdo latidens*** Agassiz, 1843 (re-assigned to †*G. eaglesomei*)

Description – The holotype of Agassiz' (1843) †*G. latidens* has a slender and symmetric root and a narrow and greatly distally inclined crown. The serrations on the mesial cutting edge

diminish in size towards the apex. Small serrations are present on the distal cutting edge, coarse serrations on the distal heel, increasing in size as the heel ascends anteriorly (Fig. 2K).

Discussion – According to Cappetta (1987), †*G. latidens* is known from the early Eocene (Ypresian, ca. 56 Ma) and is therefore considered the oldest known representative of the genus *Galeocerdo* (Purdy et al. 2001). Other records are known from middle to late Eocene deposits (Priabonian, ca. 33.9 Ma; e.g., Underwood et al. 2011).

The teeth are small and elongated (Probst 1878) and possess coarse serrations. Due to their morphological similarity to the lateral teeth of †*G. eaglesomei* (Maisch et al. 2014), Ebersole et al. (2019) stated that †*G. latidens* and †*G. eaglesomei* constitute the same species with a similar degree of monognathic heterodonty as recognised in the extant *G. cuvier*. Because of the unknown locality and horizon the †*G. latidens* holotype was collected from (Agassiz 1843), Ebersole et al. (2019) considered it to presumably constitute a *nomen dubium* and therefore merged both species into †*G. eaglesomei* instead of †*G. latidens*. The present study clearly confirms the interpretation of †*G. latidens* teeth constituting lateral teeth of †*G. eaglesomei*, hence we support the merger of both species.

†***Galeocerdo mayumbensis*** Dartevelle & Casier, 1943 (valid)

Description – Teeth of †*G. mayumbensis* are typically very compact, with thick, well-arched roots and broad crowns. The apparently anterior teeth are very erect. The distal heels are straight and the notches are weakly developed. Secondary serrations are present (Fig. 2L).

Discussion – Teeth of †*G. mayumbensis*, originally described from early Miocene deposits (Aquitanian/Burdigalian, ca. 20.44 Ma; Dartevelle and Casier 1943), occur until the late Miocene (D. J. Ward, pers. obs.). Dartevelle and Casier (1943) described the serrations to reach the apex on both cutting edges of the teeth, mentioned the similar size of the teeth to those of the modern tiger shark and emphasized distinct differences of the dentition to that of †*G.*

aduncus. However, Cigala-Fulgosi and Mori (1979) as well as Marsili et al. (2007) re-assigned †*G. mayumbensis* to †*G. aduncus*, whereas Cappetta (1987) noted that teeth of †*G. mayumbensis* are similar to the teeth of the Eocene †*G. eaglesomei*. Argyriou et al. (2015) suggested that †*G. mayumbensis* represents a valid taxon and proposed additionally to include teeth of †*G. paulinoi* in the species †*G. mayumbensis* because of their similar morphology. Andrianavalona et al. (2015) furthermore proposed to synonymize †*G. casei* with †*G. mayumbensis*. The multivariate approach in the present study clearly confirms the validity of †*G. mayumbensis* and furthermore supports the suggestion to synonymize †*G. casei* and †*G. paulinoi* with †*G. mayumbensis*.

†***Galeocerdo paulinoi*** da Silva Santos & Travassos, 1960 (re-assigned to †*G. mayumbensis)*

Description – The holotype of †*G. paulinoi* is a very compact and massive tooth, with a thick and arched root and a well curved mesial cutting edge. The notch is weakly developed and the distal cutting edge very short. It is not perceptible whether the serrations are complex or not (Fig. 2M).

Discussion – According to da Silva Santos and Travassos (1960), teeth of the Miocene (ca. 20.44 Ma) species †*G. paulinoi* are very similar to those of the extant *G. cuvier* but also resemble teeth of †*G. aduncus* and †*G. latidens*. dos Reis (2005) re-assigned †*G. paulinoi* to *G. cuvier*, whereas Argyriou et al. (2015) and Carrillo-Briceño et al. (2019) considered that the teeth possess a similar morphology to those of †*G. mayumbensis* and therefore, †*G. paulinoi* should be re-assigned to this species. We agree with Carrillo-Briceño et al. (2019) and assign †*G. paulinoi* to †*G. mayumbensis* based on shared morphological characters.

†***Galeocerdo priscus*** (Heckel, 1853) (*nomen dubium*)

Discussion – Found in the late Eocene (ca. 36 Ma) of northern Italy (Cappetta 2006), the description of this species is lacking any illustration and is based on fossil scales only (Heckel 1853). Although isolated scales may bear palaeoecological signals, they only provide very limited taxonomic information (Ferrón et al. 2014), because different morphotypes occur across the body (e.g., Ankhelyi et al. 2018; Jambura and Kriwet 2020). Consequently, we consider this species a *nomen dubium*.

†***Galeocerdo productus*** Agassiz, 1856 (re-assigned to †*P. contortus*)

Description – An illustration by Jordan (1907) depicts †*G. productus* teeth with a broad, well-arched root and a slender crown with a slightly curved mesial cutting edge. The cusp is very pointed and the serrations on the mesial cutting edge and distal heel are very minor.

Discussion – In the original description, which lacks any illustration, Agassiz (1856: p. 273) points out that teeth of this Miocene (ca. 13.8 Ma) species are very similar to those of †*G. aduncus*, only differing in "having the anterior margin of the tooth less arched, with much more minute crenulations, and the serratures on the basilar margin rather smaller". Other authors later also suggested that this species might be a synonym of †*G. aduncus* (Jordan 1907; Leriche 1908; Jordan 1910; Jordan and Gilbert 1919; Stoutamire 1975), however, this deduces from the spurious assumption of a marked heterodonty in †*G. aduncus* (see discussion of †*G. aduncus* above and †*P. contortus* below). The present study, however, shows that teeth of †*G. productus* are highly similar to teeth of †*P. contortus*, hence we merge both species and regard †*G. productus* a junior synonym of †*P. contortus*.

†***Galeocerdo pygmaeus*** (Münster, 1842) (*nomen nudum*)

Discussion – The lack of any illustration of the single Miocene (ca. 13.8 Ma) tooth referred to this species renders it difficult to identify this species unambiguously. Unfortunately, the holotype is missing, but in his original description, Münster (1842) refers to illustrations in which the tooth resembles those of †*G. aduncus*, but also shows similarities to *Acanthias* (valid: *Squalus*; Schultz 1971). Schultz (1971) therefore supposed that this tooth rather belongs to †*G. aduncus*. Münster (1846) himself re-assigned the species to †*Corax pygmaeus*. Cappetta (2006) listed †*G. pygmaeus* as a valid species, although he labelled it with a question mark and counted it to the family of megatooth sharks (Otodontidae). Due to the missing description and illustration, we regard this species a *nomen nudum*.

†***Galeocerdo rosaliaensis*** Applegate, 1978 (re-assigned to †*G. capellini*)

Description – The holotype of †*G. rosaliaensis* is very large and broad. The root of the tooth is thick, V-shaped and slightly asymmetric, the crown is broad and deeply notched distally. The mesial cutting edge and distal heel are convex and the whole crown is serrated. The secondary serration is somehow ablated but still detectable (Fig. 2N).

Discussion – †*Galeocerdo rosaliaensis* was described based on several teeth found in middle Pliocene deposits (ca. 3.6 Ma) of Mexico in 1976 (Applegate 1978). According to Applegate (1978), teeth of †*G. rosaliaensis* have about the same size and shape like those of *G. cuvier* and the only difference is that the upper half of the mesial cutting edge is flattened in †*G. rosaliaensis*. Purdy et al. (2001) suggested that †*G. rosaliaensis* is a junior synonym of †*G. capellini*, another Pliocene tiger shark species with teeth that are similar in size and shape like *G. cuvier* (see above). The present geometric morphometric study supports the assignment of †*G. rosaliaensis* to †*G. capellini* and also the validity of †*G. capellini* despite the superficial resemblance of the teeth to those of the extant *G. cuvier*.

†***Galeocerdo similis*** (Münster, 1842) (*nomen nudum*)

Discussion – †*Galeocerdo similis* from Miocene deposits (ca. 13.8 Ma) was neither described properly nor illustrated in the first description by Münster (1842). Schultz (1971) tried to re-assign the species but professed to be unable to do so as no material, no description and no illustration were locatable. Due to the missing description and illustration, we regard this species a *nomen nudum*.

†***Galeocerdo subcrenatus*** Emmons, 1858 (*nomen nudum*)

Discussion – †*Galeocerdo subcrenatus* is another tiger shark species that lacks any illustration in the original description and is apparently based on a single tooth only. The tooth is described as "nearly upright" and without distinct serrations (Emmons 1858: p. 238). According to Hay (1902: p. 312), †*G. subcrenatus* is "a doubtful species, without indicated formation". Due to the missing information, we regard this species a *nomen nudum*.

†***Galeocerdo sublaevis*** (Münster, 1842) (*nomen nudum*)

Discussion – Münster (1842) presented †*G. sublaevis* without a proper description and illustration. Later, Münster (1846) described the Miocene (ca. 13.8 Ma) teeth as being similar to those of †*G. minor* (synonymous with †*Physogaleus minor*). Giebel (1848) also described †*Physogaleus*-like characters of the teeth and Schultz (1971) presumed that the teeth belong to †*Galeorhinus affinis*. However, because an illustration and description is missing, we regard this species a *nomen nudum*.

†***Galeocerdo triqueter*** Eastman, 1904 (re-assigned to †*P. contortus*)

Description – The holotype of †*G. triqueter* has a thick and V-shaped root and a straight mesial cutting edge. The tooth is slightly inclined distally but is completely lacking a distal

notch. The cutting edges are smooth and not serrated, probably due to taphonomic processes (Fig. 2O).

Discussion – †*Galeocerdo triqueter* was described based on teeth from early/middle Miocene deposits of Maryland, USA (Eastman 1904). Purdy et al. (2001) synonymized †*G. triqueter* with *Alopias* cf. *A. vulpinus*, however, in the same work they also listed †*G. triqueter* in the synonym-list of †*G. contortus*. Cappetta (2006) suggested that the described tooth might constitute a parasymphyseal tooth of †*G. contortus* and Marsili et al. (2007) eventually synonymized †*G. triqueter* with †*G. contortus* (now valid as †*Physogaleus contortus*). We agree with Cappetta (2006) and Marsili et al. (2007) and support the assignment of †*G. triqueter* to †*P. contortus* based on shared morphological characters.

†***Physogaleus contortus*** (Gibbes, 1849) (valid)

Description – Teeth of †*P. contortus* have a prominent root but a slender cusp. The crown is slightly serrated, with the serrations on the distal heel being more pronounced. The cusp is twisted outwardly, elongated, often sigmoidal and very pointed (Fig. 2P).

Discussion – This species is known from the Eocene (ca. 36 Ma; Gibbes 1849) to Pliocene (ca. 3.6 Ma; Cicimurri and Knight 2009). Initially only known from the western Atlantic, †*Galeocerdo contortus* was considered the only endemic tiger shark species (Cappetta 1987; Müller 1999). However, Purdy et al. (2001) re-assigned †*G. acutus* from the early Oligocene (ca. 31 Ma) of Belgium and diverse teeth identified as †*G. aduncus* to †*G. contortus*. Also Reinecke et al. (2011) reported teeth of †*G. contortus* from the Miocene (ca. 18.2 Ma) of Germany. Müller (1999) pointed out that teeth of †*G. contortus* morphologically resemble teeth of †*Physogaleus* rather than those of *Galeocerdo*. Ward and Bonavia (2001) subsequently synonymized †*G. aduncus* with †*G. contortus* and referred the species to the genus †*Physogaleus*, suggesting that male sharks possessed narrow †*P. contortus*-typed teeth in the

lower jaw and broad †*G. aduncus*-typed teeth in the upper jaw (gynandric heterodonty). However, Purdy et al. (2001) stated that †*G. contortus* is a valid *Galeocerdo*-species and should not be merged with †*G. aduncus*. Also Marsili et al. (2007) agreed with Purdy et al. (2001) and added that †*G. acutus*, †*G. triqueter* and diverse teeth described as †*G. aduncus* actually belong to †*G. contortus*. Reinecke et al. (2011) finally re-assigned †*G. contortus* to †*Physogaleus contortus*, an opinion followed by Pimiento et al. (2013) and Kent (2018). We also agree with the interpreation of Reinecke et al. (2011), however, due to the long-running discussion of the genus assignment of this species, it is included here even though it doesn't belong to tiger sharks sensu stricto.

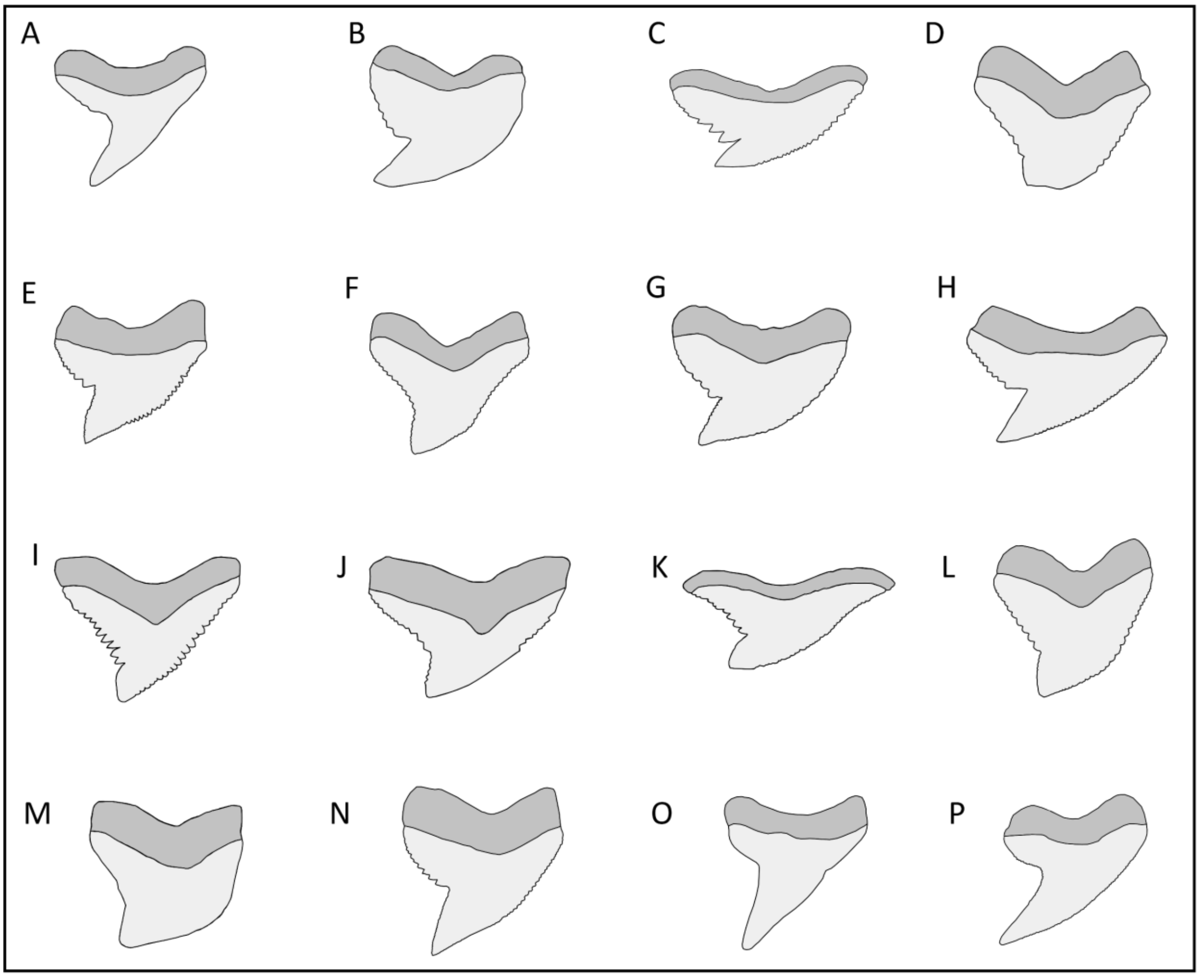

FIGURE 2: Illustration of teeth of selected extinct shark species. A, †*G. acutus*, modified after Leriche (1910); B, †*G. aduncus* holotype, modified after Agassiz (1843); C, †*G. aegyptiacus,* modified after Underwood et al. (2011); D, †*G. bigelowi*, modified after Patnaik et al. (2014); E, †*G. capellini,* modified after Lawley (1881); F, †*G. casei* holotype, modified after Müller (1999); G, †*G. clarkensis* holotype; H, †*G. davisi* holotype, modified after Davis (1888); I, †*G. eaglesomei* holotype; J, †*G. gajensis* holotype, modified after Tewari et al. (1960); K, †*G. latidens* holotype, modified after Agassiz (1843); L, †*G. mayumbensis*; M, †*G. paulinoi* holotype, modified after da Silva Santos and Travassos (1960); N, †*G. rosaliaensis* holotype; O, †*G. triqueter*; P, †*P. contortus*; All teeth are depicted in labial view. Not to scale.

Supplementary Literature

Agassiz, L. 1833–1843. Recherches sur les poissons fossiles, Vol. 3. Imprimerie de Petitpierre, Neuchâtel.

—. 1856. Notice on the fossil fishes found in California by W. P. Blake. American Journal of Science and Arts, Series 2, 21:272–275.

Andrianavalona, T. H., T. N. Ramihangihajason, A. Rasoamiaramanana, D. J. Ward, J. R. Ali, and K. E. Samonds. 2015. Miocene Shark and Batoid Fauna from Nosy Makamby (Mahajanga Basin, Northwestern Madagascar). PLoS One 10:e0129444.

Ankhelyi, M. V., D. K. Wainwritght, and G. V. Lauder. 2018. Diversity of dermal denticle structure in sharks: Skin surface roughness and three-dimensional morphology. Journal of Morphology 279:1132–1154.

Applegate, S. P. 1978. Phyletic studies; part 1, Tiger sharks. Revista mexicana de ciencias geológicas, 2:55–64.

—. 1992. The case for dignathic heterodonty in fossil specimens of the Tiger Shark genus *Galeocerdo* particularly those belonging to *G. aduncus* lineage. Abstract. Journal of Vertebrate Paleontology 12:16A–17A.

Argyriou, T., T. D. Cook, A. M. Muftah, P. Pavlakis, N. T. Boaz, and A. M. Murray. 2015. A fish assemblage from an early Miocene horizon from Jabal Zaltan, Libya. Journal of African Earth Sciences 102:86–101.

Breard, S. Q. Jr. 1991. Paleoecology of a late Eocene (Bartonian) vertebrate fauna, Moodys Branch Formation, Techeva Creek, Mississippi. Transactions of the Gulf Coast Association of Geological Societies 41:43–55.

Cappetta, H. 1987. Chondrichthyes II · Mesozoic and Cenozoic Elasmobranchii. *In* H. P. Schultze, ed. Handbook of Paleoichthyology, Vol. 3B. Gustav Fischer, Stuttgart, New York.

—. 2006. Elasmobranchii Post-Triadici. *In* W. Riegraf, ed. Fossilium Catalogus, I: Animalia. Pars 142:1–472. Backhuys Publishers, Leiden.

Carrillo-Briceño, J. D., E. Maxwell, O. A. Aguilera, R. Sánchez, and M. R. Sánchez-Villagra. 2015. Sawfishes and other Elasmobranch Assemblages from the Mio-Pliocene of the South Caribbean (Urumaco Sequence, Northwestern Venezuela). PLoS One 10:e0139230.

Carrillo-Briceño, J. D., Z. Luz, A. Hendy, L. Kocsis, O. A. Aguilera, and T. W. Vennemann. 2019. Neogene Caribbean elasmobranchs: diversity, paleoecology and paleoenvironmental significance of the Cocinetas Basin assemblage (Guajira Peninsula, Colombia). Biogeosciences 16:33–56.

Chapman, F., and G. B. Pritchard. 1904. Fossil Fish Remains from the Tertiaries of Australia. Proceedings of the Royal Society of Victoria 17:267–297.

Cicimurri, D. J., and J. L. Knight. 2009. Late Oligocene sharks and rays from the Chandler Bridge Formation, Dorchester County, South Carolina, USA. Acta Palaeontologica Polonica 54:627–647.

Cigala-Fulgosi, F., and D. Mori. 1979. Osservazioni tassonomiche sul genere *Galeocerdo* (Selachii, Carcharhinidae) con particolare riferimento a *Galeocerdo cuvieri* (Péron & Lesueur) nel Pliocene del Mediterraneo. Bollettino della Società Paleontologica Italiana 18:117–132.

Compagno, L. J. V. 1988. Sharks of the Order Carcharhiniformes. Princeton University Press, New Jersey.

da Silva Santos, R., and H. Travassos. 1960. Contribuicão à Paleontologia do Estado do Pará. Peixes Fósseis da Formacão Pirabas. Serviço Gráfico do Instituto Brasileiro de Geografia e Estatística, Rio de Janeiro.

Dames, W. B. 1883. Über eine tertiäre Wirbelthierfauna von der westlichen Insel des Birket-el-Qurun im Fajum (Aegypten). Sitzungsberichte der Königlich Preussischen Akademie der Wissenschaften zu Berlin. Pp. 129–153.

Dartevelle, E. and E. Casier. 1943. Les Poissons Fossiles du Bas-Congo et des régions voisines. Annales du Musée du Congo Belge, Sér. A (Minéralogie Géologie, Paléontologie) 3:1–200.

Davis, J. W. 1888. On Fossil Fish Remains from the Tertiary and Cretaceo-Tertiary Formations of New Zealand. Scientific Transactions of the Royal Dublin Society 4:1–48.

de Stefano, G. 1909. Osservazione sulle ittiofauna pliocenica di Orciano e San Quirico in Toscana. Bollettino della Società Geologica Italiana 28:539–648.

dos Reis, M. A. F. 2005. Chondrichthyan Fauna from the Pirabas Formation, Miocene of Northern Brazil, with Comments on Paleobiogeography. Anuário do Instituto de geociências 28:31–58.

Eastman, C. R. 1904. Pisces (of Miocene of Maryland). Maryland Geological Survey 2:71–93.

Ebersole, J. A., D. J. Cicimurri, and G. L. Stringer. 2019. Taxonomy and biostratigraphy of the elasmobranchs and bony fishes (Chondrichthyes and Osteichthyes) of the lower-to-middle Eocene (Ypresian to Bartonian) Claiborne Group in Alabama, USA, including an analysis of otoliths. European Journal of Taxonomy 585:1–274.

Emmons, E. 1858. Report of the North-Carolina Geological Survey. Agriculture of the eastern countries; together with descriptions of the fossils of the Marl Beds. H. D. Turner, Raleigh, North Carolina.

Ferrón, H., C. Pla, C. Martínez-Pérez, M. J. Escudero-Mozo, and H. Botella. 2014. Morphometric Discriminant Analysis of isolated chondrichthyan scales for palaeoecological inferences: the Middle Triassic of the Iberian Chain (Spain) as a case of study. Journal of Iberian Geology 40:87–97.

Gibbes, R. W. 1849. Monograph of the fossil Squalidae of the United States. Journal of the Academy of Natural Sciences of Philadelphia 1:191–206.

Giebel, C. G. 1848. Fauna der Vorwelt, mit steter Berücksichtigung der lebenden Thiere. Erster Band: Wirbelthiere. Dritte Abtheilung: Fische. Brockhaus, Leipzig.

Hay, O. P. 1902. Bibliography and Catalogue of the Fossil Vertebrata of North America. Bulletin of the United States Geological and Geographical Survey of the Territories 179:1–868.

Heckel, J. 1853. Über fossile Fische aus dem Chiavon und das geologische Alter der sie enthaltenden Schichten. Sitzungsberichte der Kaiserlichen Akademie der Wissenschaften. Mathematisch-Naturwissenschaftliche Klasse 11:322–334.

Hooijer, D. A. 1954. Pleistocene Vertebrates from Celebes. IX. Elasmobranchii. Proceedings Koninklijke Nederlandse Akademie Van Wetenshappen, Series B (Physical Sciences) 57:475–485.

Jambura, P. L., C. Pfaff, C. J. Underwood, D. J. Ward, and J. Kriwet. 2018. Tooth mineralization and histology patterns in extinct and extant snaggletooth sharks, *Hemipristis* (Carcharhiniformes, Hemigaleidae)–Evolutionary significance or ecological adaptation? PLoS One 13:e0200951.

Jambura, P. L., and J. Kriwet. 2020. Articulated remains of the extinct shark *Ptychodus* (Elasmobranchii, Ptychodontidae) from the Upper Cretaceous of Spain provide insights into gigantism, growth rate and life history of ptychodontid sharks. PLoS One 15:e0231544.

Jordan, D. S. 1907. The fossil fishes of California with supplementary notes on other species of extinct fishes. University of California Publications. Bulletin of the Department of Geology 5:95–144.

—. 1910. Notes on ichthyology. The American Naturalist 44:178–191.

Jordan, D. S., and J. Z. Gilbert. 1919. Fossil Fishes of Southern California. II. Fossil Fishes of the Miocene (Monterey) Formations. Leland Stanford Junior University Publications, University Series 38:13–60.

Kent, B. W. 2018. The Cartilaginous Fishes (Chimaeras, Sharks, and Rays) of Calvert Cliffs, Maryland, USA. Pp. 45–157 *in* S. J. Godfrey, ed. The Geology and Vertebrate Paleontology of Calvert Cliffs, Maryland, USA.

Lawley, R. 1876. Nuovi studi sopra ai pesci ed altri vertebrati fossili delle colline toscane. Tipografia dell’arte della Stampa, Florence.

—. 1881. Studi comparativi sui pesci fossili coi viventi dei generi *Carcharodon*, *Oxyrhina* e *Galeocerdo*. Tipografia T. Nistri e C.

Leriche, M. 1908. Observations sur les Squales néogènes de la Californie. Annales de la Société Géologique du Nord 37:302–306.

—. 1910. Note sur les Poissons stampiens du Bassin de Paris. Annales de la Société Géologique du Nord 39:324–336.

Maisch, H. M., M. A. Becker, B. W. Raines, and J. A. Jr. Chamberlain. 2014. Chondrichthyans from the Tallahatta-Lisbon Formation contact (middle Eocene), Silas, Choctaw County, Alabama. Paludicola 9:183–209.

Malyshkina, T. P., G. Gonzáles-Barba, and A. F. Bannikov. 2013. Records of elasmobranchian teeth in the Bartonian of the northern Caucasus (Russia) and Crimea (Ukraine). Paleontological Journal 47:98–103.

Manning, E. 1990. The late early Miocene Sabine River. *In* E. Kinsland and T. Cagle, eds. Transactions of the Gulf Coast Association of Geological Societies 40:531–549.

Marsili, S., G. Carnevale, E. Danese, G. Bianucci, and W. Landini. 2007. Early Miocene vertebrates from Montagna della Maiella, Italy. Annales de Paléontologie 93:27–66.

Mehrotra, D. K., V. P. Mishra, and S. Srivastava. 1973. Miocene sharks from India. Recent Researches in Geology 1:180–200.

Moyer, J. K., and W. E. Bemis. 2017. Shark teeth as edged weapons: serrated teeth of three species of selachians. Zoology 120:101–109.

Müller, J., and F. G. J. Henle. 1837. Gattungen der Haifische und Rochen nach einer von ihm mit Hrn. Henle unternommenen gemeinschaftlichen Arbeit über die Naturgeschichte der Knorpelfische. Berichte der Königlichen Preussischen Akademie der Wissenschaften zu Berlin 2:111–118.

Müller, A. 1999. Ichthyofaunen aus dem atlantischen Tertiär der USA. Leipziger Geowissenschaften.

Münster, G. G. 1842. Beschreibung einiger fossilen Fischzähne aus dem Tertiär-Becken von Wien. Beiträge zur Petrefacten-Kunde 5:65–69.

—. 1846. Ueber die in der Tertiär-Formation des Wiener Beckens vorkommenden Fisch-Ueberreste, mit Beschreibung einiger neuen merkwürdigen Arten. Beiträge zur Petrefacten-Kunde **7**:1–31.

Parmley, D., and D. J. Cicimurri. 2003. Late Eocene Sharks of the Hardie Mine local fauna of Wilkinson County, Georgia. Georgia Journal of Science 61:153–179.

Patnaik, R., K. M. Sharma, L. Mohan, B. A. Williams, R. Kay, and P. Chatrath. 2014. Additional vertebrate remains from the early Miocene of Kutch, Gujarat. Special Publication of the Paleontological Society of India 5:335–351.

Péron, F., and C. A. Lesueur. 1822. Description of a Squalus, of a very large size, which was taken on the coast of New Jersey. Journal of the Academy of Natural Sciences of Philadelphia 2:343–352.

Pimiento, C., G. Gonzales-Barba, A. J. W. Hendy, C. Jaramillo, B. J. MacFadden, C. Montes, S. C. Suarez, and M. Shippritt. 2013. Early Miocene chondrichthyans from the Culebra

Formation, Panama: a window into marine vertebrate faunas before closure of the Central American Seaway. Journal of South American Earth Sciences 42:159–170.

Probst, J. 1878. Beiträge zur Kenntniss der fossilen Fische aus der Molasse von Baltringen. Hayfische. Jahreshefte des Vereins für vaterländische Naturkunde in Württemberg 34:113–154.

Purdy, R. W. 1998. The early Miocene fish fauna from the Pollack Farm site, Delaware. *In* R. N. Benson, ed. Geology and Paleontology of the lower Miocene Pollack Farm Fossil Site, Delaware. Newark: Delaware Geological Survey, Special Publication 22:133–139.

Purdy, R. W., V. P. Schneider, S. P. Applegate, J. H. McLellan, R. L. Meyer and B. H. Slaughter. 2001. The neogene sharks, rays, and bony fishes from Lee Creek Mine, Aurora, North Carolina. Smithsonian Contributions to Paleobiology 90:71–202.

Reinecke, T., S. Louwye, U. Havekost, and H. Moths. 2011. The elasmobranch fauna of the late Burdigalian, Miocene, at Werder-Uesen, Lower Saxony, Germany, and its relationship with early Miocene faunas on the North Atlantic, Central Paratethys and Mediterranean. Palaeontos 20:1-170.

Samonds, K. E., T. H. Andrianavalona, L. A. Wallett, I. S. Zalmout, and D. J. Ward. 2019. A middle-late Eocene neoselachian assemblage from nearshore marine deposits, Mahajanga Basin, northwestern Madagascar. PLoS One 14:e0211789.

Sarangdhar, P. N. 1943. Tiger shark – *Galeocerdo tigrinus* Müller and Henle. Feeding and Breeding Habits. Journal of the Bombay Natural History Society 44:102–110.

Schultz, O. 1971. Die Selachier-Fauna (Pisces, Elasmobranchii) des Wiener Beckens und seiner Randgebiete im Badenien (Miozän). Annalen des Naturhistorischen Museums in Wien 75:311–341.

Snodgrass, R. E., and E. Heller. 1905. Papers from the Hopkins-Stanford Galapagos Expedition, 1898-1899. XVII. Shore fishes of the Revillagigedo, Clipperton, Cocos and Galapagos islands. Proceedings of the Washington Academy of Sciences 6:333–427.

Storms, R. 1894. Troisième note sur les poissons du terrain rupélien. Bulletin de la Société Belge de Géologie, de Paléontologie et d'Hydrologie 8:67–82.

Stoutamire, S. 1975. A new Middle Miocene Vertebrate Fauna from the Florida Panhandle. MSc Thesis. Texas Tech University, USA.

Stromer, E. 1903. Haifischzähne aus dem unteren Mokattam bei Wasta in Egypten. Neues Jahrbuch für Mineralogie, Geologie und Paläontologie 1:29–41.

—. 1905. Die Fischreste des mittleren und oberen Eocäns von Ägypten. I. Teil: Selachii, B. Squaloidei, und II. Teil: Telestomi, A. Ganoidei. Beiträge zur Paläontologie und Geologie Österreich–Ungarns 18:163–185.

Sweydan, S., G. Merzeraud, E. M. Essid, W. Marzougui, R. Temani, H. K. Ammar, L. Marivaux, M. Vianey-Liaud, R. Tabuce, and S. Adnet. 2019. A reworked elasmobranch fauna from Tunisia providing a snapshot of Eocene-Oligocene Tethyan faunas. Journal of African Earth Sciences 149:194–206.

Tewari, B. S., M. N. Chaturvedi, and M. P. Singh. 1960. Two new Species of Shark Teeth from Gaj Beds of Matanumarh, Kutch. Journal of Palaeontological Society of India 9:74–76.

Underwood, C. J., D. J. Ward, C. King, S. M. Antar, I. S. Zalmout, and P. D. Gingerich. 2011. Shark and ray faunas in the Middle and Late Eocene of the Fayum Area, Egypt. Proceedings of the Geologists' Association 122:47–66.

Van Beneden, P. J. 1873. Paléontologie des vertébrés. Pp. 353–388 *in* M. E. Van Bemmel, ed. Patria Belgica, Encyclopédie nationale ou exposé méthodique de toutes les connaissances relatives à la Belgique ancienne et modern, physique, sociale et intellectuelle 1. Bruylant-Christophe and Cie, Bruxelles.

Ward, D. J., and C. G. Bonavia. 2001. Additions to, and a review of, the Miocene shark and ray fauna of Malta. Central Mediterranean Naturalist 3:131–146.

Westgate, J. W. 1984. Lower Vertebrates from the Late Eocene Crow Creek Local Fauna, St. Francis County, Arkansas. Journal of Vertebrate Paleontology 4:536–546.

—. 1989. Lower Vertebrates from an estuarine facies of the middle Eocene Laredo Formation (Claiborne Group), Webb County, Texas. Journal of Vertebrate Paleontology 9:282–294.

White, E. I. 1955. Notes on African Tertiary Sharks. Bulletin of the Geological Survey of Nigeria 5:319–325.

—. 1956. The Eocene Fishes of Alabama. Bulletins of American Paleontology 36:123–150.

Woodward, A. S. 1889. Catalogue of the Fossil Fishes in the British Museum. Part I. Containing the Elasmobranchii. Trustees of the British Museum (Natural History).
