# Supplementary material for: Evolution, diversity, and disparity of the tiger shark lineage Galeocerdo in deep time
Source: Paleobiology. Author manuscript; Available in PMC 2021 Dec 2. (PMC7612061; doi:10.1017/pab.2021.6)
Supplement: Supplementary data [file EMS135981-supplement-Supplementary_data.zip › R_CODE.docx]

######################################################

# Source Code: Türtscher et al. #

# Evolution, diversity and disparity of the tiger #

# shark lineage Galeocerdo in deep time #

# #

# Paleobiology #

######################################################

### set working directory

setwd("/working_directory")

### load the packages and data

library(geomorph) # includes RRPP

Gal <- readland.tps("Galeocerdo.tps",

specID = "imageID",

readcurves = TRUE, warnmsg = TRUE)

Gal.Class <- read.csv("Classifiers_Galeocerdo.csv",

header = TRUE, sep = ",")

Gal.Cv <- as.matrix(read.csv("Sliders_Galeocerdo.csv",

header = TRUE))

######################################################

### Generalized Procrustes Analysis (GPA)

Gal.GPA <- gpagen(Gal, curves = Gal.Cv, ProcD = FALSE,

print.progress = FALSE)

######################################################

### Principal Component Analysis (PCA)

Gal.PCA <- gm.prcomp(Gal.GPA$coords)

summary(Gal.PCA) # shows the PC axes

######################################################

# Genus comparison reported in Table 1 (ANOVA)

# and Table 2 (pairwise comparisons)

# data frame with shape data

Gal.gdf <- geomorph.data.frame(shape = Gal.GPA$coords,

Gen = Gal.Class$Genus,

Sp = Gal.Class$Species)

### Procrustes ANOVA

Gal.aov <- procD.lm(shape~Gen, data = Gal.gdf, iter = 999,

RRPP = TRUE, print.progress = FALSE)

summary(Gal.aov)

# pairwise comparisons

Gen.PW <- pairwise(Gal.aov, groups = Gal.gdf$Gen,

print.progress = FALSE)

summary(Gen.PW, test.type = "dist", confidence = 0.95,

stat.table = TRUE)

######################################################

# for further analyses on tiger shark species,

# Hemipristis and Phyosgaleus won‘t be included

Gal.Galeo <- Gal.GPA$coords[,,c(29:87,90,93:95,98:236,

238:276,308:324,326:327,

329:358,362:367,372:396,

399:420,424,428:433,

435:444,460:462,468:486,

488:513,535:569)]

# only these specimens (= Galeocerdo) from the data

# (= Gal.Class) will be included

Galeo.Class <- Gal.Class[c(29:87,90,93:95,98:236,

238:276,308:324,326:327,

329:358,362:367,372:396,

399:420,424,428:433,

435:444,460:462,468:486,

488:513,535:569),]

# subset including only Galeocerdo

Galeo.sub <- subset(Galeo.Class, Genus == "Galeo",

select = c("Specimen", "Epoch", "Species"))

# data frame with shape data

Galeo.gdf <- geomorph.data.frame(shape = Gal.Galeo,

Ep = Galeo.sub$Epoch,

Sp = Galeo.sub$Species)

######################################################

# Epoch comparison

# Procrustes ANOVA (Table 1)

Gal.Ep.aov <- procD.lm(shape~Ep, data = Galeo.gdf,

iter = 999,

RRPP = TRUE, print.progress = FALSE)

summary(Gal.Ep.aov)

# pairwise comparisons (Table 2)

Ep.PW <- pairwise(Gal.Ep.aov, groups = Galeo.gdf$Ep)

summary(Ep.PW, test.type = "dist", confidence = 0.95,

stat.table = TRUE)

######################################################

# Species comparison

# exclude species without replicates

Sp.Class.rep <- Galeo.sub[-c(60,62,63,67,68,266,389),]

# Procrustes ANOVA (Table 1)

Gal.Sp.aov <- procD.lm(shape~Sp, data = Galeo.gdf,

iter = 999, RRPP = TRUE,

print.progress = FALSE)

summary(Gal.Sp.aov)

# pairwise comparisons (Table 2)

Sp.PW <- pairwise(Gal.Sp.aov, groups = Galeo.gdf$Sp)

summary(Sp.PW, test.type = "dist", confidence = 0.95,

stat.table = TRUE)

######################################################

# Disparity through time (Table 3)

Disparity <- morphol.disparity(shape~Ep, groups = Galeo.gdf$Ep,

data = Galeo.gdf, iter = 999)

Disparity

# show barplot

barplot(Disparity$Procrustes.var)

######################################################

# #

# PLOTS #

# #

######################################################

# define the PC axes

Gal.Class$PC1 <- as.matrix(Gal.PCA$x[,1])

Gal.Class$PC2 <- as.matrix(Gal.PCA$x[,2])

### Plot Example: Epochs

# define the groups

Galeo <- Gal.Class[Gal.Class$Genus == "Galeo",]

Eoc <- Galeo[Galeo$Epoch == "Eocene",]

Mio <- Galeo[Galeo$Epoch == "Miocene",]

Oli <- Galeo[Galeo$Epoch == "Oligocene",]

Hol <- Galeo[Galeo$Epoch == "Holocene",]

Pli <- Galeo[Galeo$Epoch == "Pliocene",]

Phys <- Gal.Class[Gal.Class$Genus == "Physo",]

Hemi <- Gal.Class[Gal.Class$Genus == "Hemi",]

# load the colour palette

library(viridisLite) # includes viridis and inferno

# create the amount of colours you need

n <- 4

g4 <- inferno(n)

g4

### scatterplot

plot(Gal.Class$PC2 ~ Gal.Class$PC1, data = Gal.Class,

type = "n", xlab = "PC1", ylab = "PC2")

abline(v = 0, col = "gray77", lty = 2) # adds straight lines through plot

abline(h = 0, col = "gray77", lty = 2)

points(Gal.Class$PC1, Gal.Class$PC2, pch = 19, col = "gray75",

cex = 1)

points(Phys$PC1, Phys$PC2, pch = 19, col = "gray83", cex = 1)

points(Hemi$PC1, Hemi$PC2, pch = 19, col = "gray83", cex = 1)

points(Eoc$PC1, Eoc$PC2, pch = 21, col = "black", lwd = 0.5,

bg = "#781C6DFF", cex = 1.5)

points(Oli$PC1, Oli$PC2, pch = 22, col = "black", lwd = 0.5,

bg = "#2D708EFF", cex = 1.5)

points(Mio$PC1, Mio$PC2, pch = 21, col = "black", lwd = 0.5,

bg = "#DD513AFF", cex = 1.5)

points(Pli$PC1, Pli$PC2, pch = 21, col = "black", lwd = 0.5,

bg = "#FCA50AFF", cex = 1.5)

points(Hol$PC1, Hol$PC2, pch = 21, col = "black", lwd = 0.5,

bg = "#FCFFA4FF", cex = 1.5)

######################################################

### show the mean shapes of groups

# define links between landmarks

Gal.Links <- define.links(Gal[,,4], ptsize = 1,

links = NULL)

# estimate mean shape for a set of aligned specimens

Ref <- mshape(Gal.GPA$coords)

# Plot shape differences btw. a Reference and target

# specimen (e.g. min, max of PC1)

plotRefToTarget(Ref, Gal.PCA$shapes$shapes.comp1$min,

method = "point", links = Gal.Links)

# extract the mean shape of each group

mean.shape <- mshape(Gal.GPA$coords)

Mshape.gdf <- geomorph.data.frame(shape = Gal.GPA$coords,

species = Gal.Class$Shapespec,

genus = Gal.Class$Genus,

epoch = Gal.Class$Epoch)

# group means

Gal.Ep.shape <- procD.lm(shape~epoch, data = Mshape.gdf,

print.progress = FALSE)

PCAM <- prcomp(Gal.Ep.shape$fitted)

means = unique(round(PCAM$x,20))

means

x <- Gal.Ep.shape$X

x

Y <- x[,-1]

Y

# interceptions

Eocene <- c(0,0,0,0)

Holocene <- c(1,0,0,0)

Miocene <- c(0,1,0,0)

Oligocene <- c(0,0,1,0)

Pliocene <- c(0,0,0,1)

# Shape predictions from numeric predictors

preds.Epochs <- shape.predictor(arrayspecs(Gal.Ep.shape$fitted,67,2),

x = Y,

Intercept = TRUE,

Eocene = Eocene,

Holocene = Holocene,

Miocene = Miocene,

Oligocene = Oligocene,

Pliocene = Pliocene)

# compare a group with reference mean shape

plotRefToTarget(mean.shape, preds.Epochs$Pliocene, links = Gal.Links,

method = "point")

# show a group without reference mean shape

plotRefToTarget(preds.Epochs$Eocene, preds.Epochs$Eocene,

links = Gal.Links,

method = "point", label = FALSE,

gridPars = gridPar(pt.bg = "#781C6DFF",

pt.size = 2.5))

# create deformation grids

plot(Gal.PCA, axis1 = 1, axis2 = 2, pch = 21, col = "black",

bg = as.numeric(Gal.Class$Epoch))

picknplot.shape(plot(Gal.PCA), method = "TPS")
